# A novel lipid metabolism gene signature for clear cell renal cell carcinoma using integrated bioinformatics analysis

**DOI:** 10.3389/fcell.2023.1078759

**Published:** 2023-02-14

**Authors:** Ke Li, Yan Zhu, Jiawei Cheng, Anlei Li, Yuxing Liu, Xinyi Yang, Hao Huang, Zhangzhe Peng, Hui Xu

**Affiliations:** ^1^ Department of Nephrology, Xiangya Hospital, Central South University, Changsha, China; ^2^ Department of Urology, Xiangya Hospital, Central South University, Changsha, China; ^3^ National Clinical Research Center for Geriatric Disorders, Xiangya Hospital, Central South University, Changsha, China; ^4^ Foreign Languages Institute, China University of Geosciences Wuhan, Wuhan, China; ^5^ Hunan Key Laboratory of Organ Fibrosis, Central South University, Changsha, China; ^6^ Department of Cell Biology, School of Life Sciences, Central South University, Changsha, China

**Keywords:** clear cell renal cell carcinoma, lipid metabolism genes, differentially expressed genes, prognostic genes, single-cell analysis, early diagnosis

## Abstract

**Background:** Clear cell renal cell carcinoma (ccRCC), which is the most prevalent type of renal cell carcinoma, has a high mortality rate. Lipid metabolism reprogramming is a hallmark of ccRCC progression, but its specific mechanism remains unclear. Here, the relationship between dysregulated lipid metabolism genes (LMGs) and ccRCC progression was investigated.

**Methods:** The ccRCC transcriptome data and patients’ clinical traits were obtained from several databases. A list of LMGs was selected, differentially expressed gene screening performed to detect differential LMGs, survival analysis performed, a prognostic model established, and immune landscape evaluated using the CIBERSORT algorithm. Gene Set Variation Analysis and Gene set enrichment analysis were conducted to explore the mechanism by which LMGs affect ccRCC progression. Single-cell RNA-sequencing data were obtained from relevant datasets. Immunohistochemistry and RT-PCR were used to validate the expression of prognostic LMGs.

**Results:** Seventy-one differential LMGs were identified between ccRCC and control samples, and a novel risk score model established comprising 11 LMGs (*ABCB4*, *DPEP1*, *IL4I1*, *ENO2*, *PLD4*, *CEL*, *HSD11B2*, *ACADSB*, *ELOVL2*, *LPA*, and *PIK3R6*); this risk model could predict ccRCC survival. The high-risk group had worse prognoses and higher immune pathway activation and cancer development.

**Conclusion:** Our results showed that this prognostic model can affect ccRCC progression.

## 1 Introduction

The Global Cancer Statistics of 2020 revealed that more than 431,000 individuals were diagnosed with primary renal carcinoma, with more than 179,000 of these individuals dying (Sung et al., 2021). Renal cell carcinoma (RCC) comprises a group of malignant tumors that originate in nephrons. In 2022, 79,000 new cases and 13,920 deaths associated with RCC was reported in the United States (Siegel et al., 2022). Clear cell RCC (ccRCC) is the most prevalent type of RCC (Humphrey et al., 2016; Obradovic et al., 2021) and it is not susceptible to chemoradiotherapy (Hsieh et al., 2017). Antiangiogenic agents that target the vascular endothelial growth factor pathway, inhibitors of the mammalian target of rapamycin (mTOR) pathway, and immunotherapy with programmed cell death one pathway blockers have all been shown to improve disease control (Atkins and Tannir, 2018). Unfortunately, the current five-year survival rate for patients with advanced ccRCC is only 10% (Sanchez-Gastaldo et al., 2017), which is more than 90% lower than that of patients without metastases. Therefore, the recognition of biomarkers for early diagnosis would be of great clinical significance. With the emergence of omics technologies such as genomics and imaging, multi-omics analysis of urinary tract tumors has become a reliable way to effectively search for prognostic assessment molecules and potential therapeutic targets (Ferro et al., 2022). Several prognostic molecules have been identified through genomic analysis of kidney cancer, among which *BAP1* mutations have been suggested to be associated with a lower ccRCC survival rate (Motzer et al., 2020).

Lipids are important biomolecules that are diverse and have complex structures; these structures determine the diversity and complexity of their functions. The tumorigenic effects and underlying mechanisms of lipid accumulation common in many cancers are still poorly understood, but there are many studies reporting a link between lipids and renal cancer. The most common subtype of RCC is ccRCC, which is characterized by lipid-rich cytoplasmic deposits (Shen et al., 2021), and all types of RCC are associated with reprogramming of fatty acid (FA) metabolism (Chakraborty et al., 2021). In RCC, lipid synthesis and metabolism are significantly altered. Inhibition of FA metabolism promotes lipid deposition in ccRCC and cancer progression (Du et al., 2017). Meanwhile, inactivation of the AMPK-GATA3-ECHS1 pathway in ccRCC can promote FA synthesis and tumor cell growth (Qu et al., 2020). In RCC, the expression of enzymes involved in lipid metabolism was also altered. Transcription factor E2F1 is overexpressed in RCC and can promote the expression of lipogenic enzymes, thereby promoting tumor growth and metastasis (Shen et al., 2021). In addition, inhibitors of FA synthase inhibited the growth and invasion of renal cancer cells (Horiguchi et al., 2008). Many FA-related proteins have been reported to be closely related with ccRCC, such as FABP7 (Nagao et al., 2018) and FATP4 (Kim et al., 2019).

In the hypoxic, acidic, and nutrient-deficient tumor microenvironment (TME), cancer and immune cells tend to use lipids as a source of energy and signaling molecules. In TME, lipids are a double-edged sword that can support both antitumor and pro-tumor immune responses (Yu W et al., 2021). Lipid deposition and reprogramming of lipid metabolism are common in the TME of RCC. Lipids can affect both tumor and immune cells (Xia et al., 2021). FAs are directly involved in the signaling of immune cells, thereby regulating their function (Xia et al., 2021). High cholesterol can disrupt the lipid metabolism network in T cells, thus exerting an immune suppression function (Li et al., 2003). High cholesterol expression in tumor cells can protect them from immune surveillance and other treatments (Xia et al., 2021). Therefore, more attention should be focused on the changes in immune infiltration caused by lipid metabolism in RCC.

This study attempted to develop a prognostic model based on lipid metabolism genes (LMGs) to predict patient survival in The Cancer Genome Atlas-Kidney Renal Clear Cell Carcinoma (TCGA-KIRC) dataset. The results were then validated using four independent datasets, including integrated single-cell RNA-sequencing data (scRNA-Seq) from the Gene Expression Omnibus (GEO) database. This study not only aimed to reveal the relationship between lipid metabolism changes and ccRCC pathogenesis, but also to determine the molecular mechanism and provide insights into novel therapeutic targets for ccRCC treatments.

## 2 Materials and methods

### 2.1 Data collection and single-cell RNA-sequencing data processing

**FIGURE 1:**
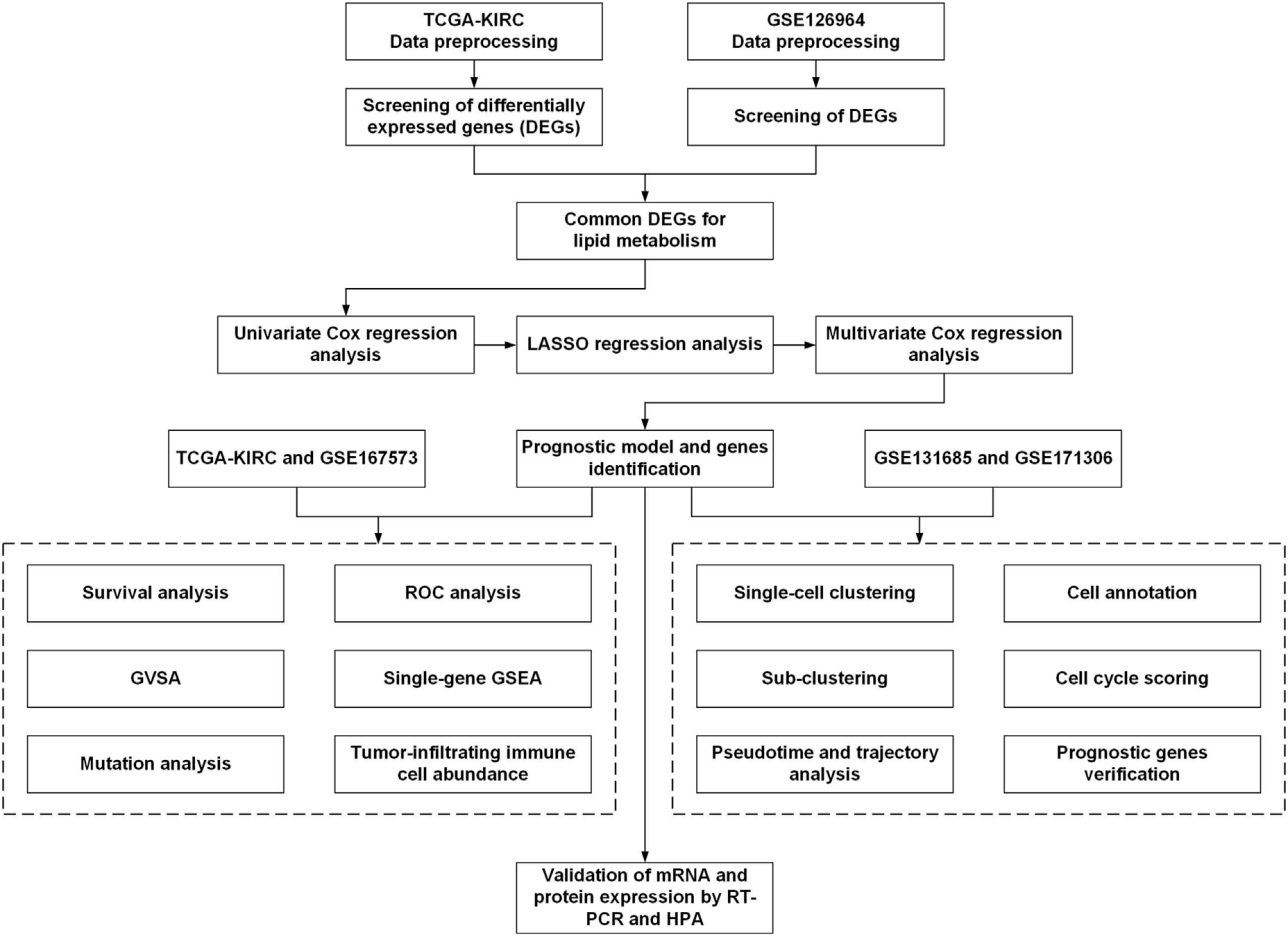
Schematic diagram of the workflow of the present study.

The workflow for this current study is presented in Figure 1. The gene expression RNA-Seq datasets GSE126964 (Zhao et al., 2020) and GSE167573 (Sun et al., 2021), were selected and downloaded from the GEO database (https://www.ncbi.nlm.nih.gov/geo/). The expression matrix was annotated with gene symbols using information from the GPL20795 and GPL20795 platform files. GSE126964 contained 55 ccRCC tumor tissues and 11 matched normal tissues, and GSE167573 contained 63 ccRCC tumor tissues and 14 adjacent normal tissues. All data were processed using R (version 4.0.4) and RStudio (version 1.2.5033).

TCGA-KIRC RNA-Seq data (both TPM and count data) and associated clinical information were obtained from the University of California, Santa Cruz (UCSC) browser Xena (http://xena.ucsc.edu/).

The scRNA-Seq data from GSE131685 (Liao et al., 2020) and GSE171306 (Yu Z et al., 2021) were downloaded from the GEO database and processed using the R package “Seurat” (version 4.0.2) (Hao et al., 2021). Three healthy kidney samples from GSE131685 and two ccRCC samples from GSE171306 were merged for further analysis. The scRNA-Seq data were processed following a previously described method (Huang et al., 2021). Cell clusters were annotated manually using the R package “SingleR” (version 1.4.1) and previously published results. The *t*-distributed stochastic neighbor (*t*-SNE) algorithm was used to explore and visualize cluster classifications across cell samples. Trajectory and pseudotime analyses of ccRCC tumor cells were performed using the R package “monocle” (Qiu et al., 2017).

### 2.2 Differentially expressed gene (DEG) identification

The “limma” R package (version 3.48.0) (Ritchie et al., 2015) was used to identify DEGs between ccRCC and normal samples in data from the GSE126964 and TCGA-KIRC datasets. The cut-off criteria (adjusted *p*-value and |log_2_fold change|) were set as <0.05 and ≥2.0, respectively. Heatmap plots were generated using the R package “pheatmap” (version 1.0.12).

### 2.3 Univariate Cox, least absolute shrinkage and selection operator (LASSO), and multivariate Cox regression analyses

Univariate Cox regression analysis was performed to screen differential LMGs that were significantly associated with overall survival (OS) in the TCGA-KIRC dataset. Genes with *p* <0.1 were included in subsequent research.

The LASSO regression analysis was performed using the R package “glmnet” (version 4.1–2). The independent variable in the regression was the normalized expression matrix of candidate differential LMGs; response variables were OS and patient status in the TCGA-KIRC cohort. Then, multivariate Cox regression model analysis was performed to establish a Cox proportional hazard regression prognostic model. The risk score was determined using the following formula:

$$Risk\;Score=\overset{n}{\underset{i=1}{\sum }}\beta_{i}\times {\mathrm{Exp}}_{i}$$

where *β* designates the regression coefficient and *Exp* designates the expression levels of each lipid metabolism gene, *i*., Samples in the TCGA-KIRC cohort were divided into high- or low-risk groups according to their median risk scores. Receiver operating characteristic (ROC) and Kaplan-Meier analyses were conducted between the high- and the low-risk groups.

### 2.4 Mutation analysis

The R package “maftools” (version 2.6.05) was used to calculate the tumor mutation burden score for each sample from high- and low-risk groups and to generate the oncoplot waterfall plot.

### 2.5 Analysis of tumor-infiltrating immune cell abundance

The CIBERSORT algorithm (https://cibersort.stanford.edu/) (Newman et al., 2015) was used to assess the proportions of 22 types of infiltrating immune cells, based on the TCGA-KIRC dataset. Wilcoxon signed-rank tests were used to compare these 22 types of immune cells between groups; the R package “ggplot2” (version 3.3.5) was used for visualization. Correlation analysis of the relationship between risk score and immune cells was visualized using the “corrplot” R package (version 0.92).

### 2.6 Biological function prediction

Kyoto Encyclopedia of Genes and Genomes (KEGG) analysis was conducted on the high- and low-risk groups *via* Gene Set Variation Analysis (GSVA). Reference information was downloaded from the Molecular Signature Database v7.4 (MSigDB v7.4, http://software.broadinstitute.org/gsea/msigdb/index.jsp) (Hanzelmann et al., 2013). Enriched pathways with false discovery rates of <0.05 were considered statistically significant.

Gene set enrichment analysis (GSEA) was used to detect potential molecular mechanisms of the prognostic model. Enriched terms that were predicted to be associated with the KEGG pathway in c2.cp.v7.2.symbols.gmt and Gene Ontology (GO) terms in c5.all.v7.2.symbols.gmt were screened using GSEA. A *p*-value of <0.05 was considered statistically significant.

### 2.7 Cell culture

The ccRCC 786-o and human embryonic kidney HEK293 cell lines were purchased from the Cell Lab of Central South University. The cell lines were maintained in Dulbecco’s Modified Eagle’s Medium with high glucose (Procell Life Science and Technology Co., Ltd., Wuhan, China) and 10% fetal bovine serum (Procell Life Science and Technology Co., Ltd.). Cells were maintained at 37°C in a humidified incubator with 5% CO_2_.

### 2.8 RNA isolation and RT-PCR

Total RNA of cell samples was extracted using the TRIzol reagent (Solarbio, Beijing, China) and subjected to reverse transcription with random primers using the RevertAid First Strand cDNA Synthesis Kit (Thermo Fisher Scientific, United States). The expression level of targeted genes was measured with the Maxima SYBR Green/ROX qPCR Mix (Thermo Fisher Scientific, United States) using a real-time PCR system (Roche, Basel, Switzerland). Relative RNA expression levels were calculated using the 2^(−△△CT)^ method and U6 as an internal control. Primer sequences will be provided upon request.

### 2.9 Protein expression level analysis in the human protein atlas (HPA) database

Immunohistochemistry images of ccRCC and normal renal samples were obtained from the HPA database (https://www.proteinatlas.org/).

### 2.10 Statistical analysis

Statistical analyses were performed with GraphPad Prism (version 8.0) using Student’s *t* test. Data were considered significant when *p ≤ 0.05, **p ≤ 0.01, or ***p ≤ 0.001.

## 3 Results

### 3.1 Expression profile of LMGs

**FIGURE 2:**
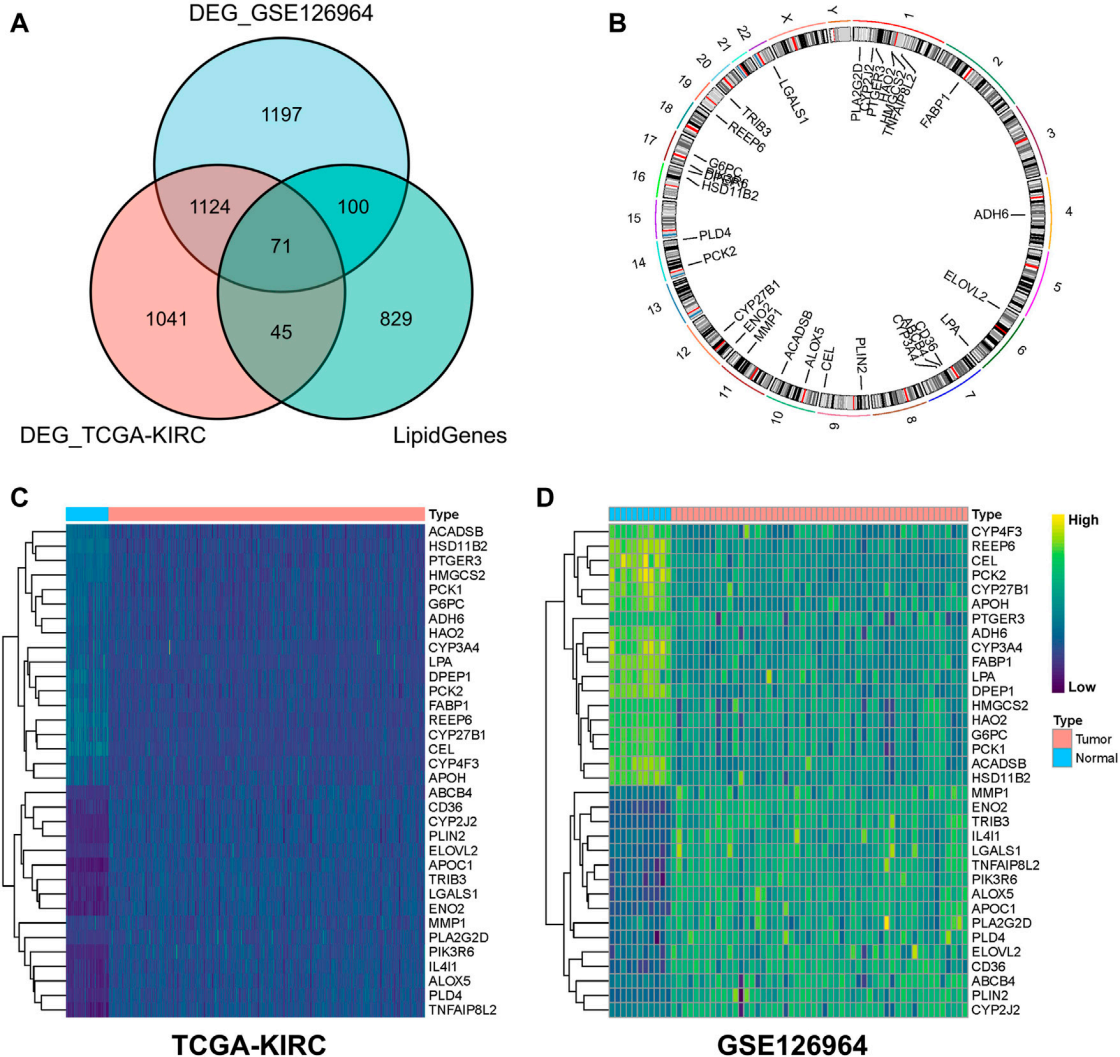
Screening for differential lipid metabolism genes (LMGs). **(A)** Venn diagram of common differential LMGs between GSE126964 and TCGA-KIRC datasets. **(B)** Chromosomal locations of LMGs. Heatmap illustrating LMG expression differences between clear cell renal cell carcinoma (ccRCC) and normal tissues in **(C)** TCGA-KIRC and **(D)** GSE126964 databases.

In total, 1,045 LMGs were selected (Supplementary Table S1) based on previous studies (Li et al., 2020). Intersection analysis of the DEGs in TCGA-KIRC and GSE126964 datasets screened out a total of 71 differential LMGs (Figure 2A). Of these LMGs, univariate Cox regression analysis identified thirty-four OS-related LMGs (*p* < 0.1); part of these LMGs and their chromosomal locations are summarized in Figure 2B and Supplementary Table S2.

In the TCGA-KIRC and GSE126964 databases, LMG expression profiles of ccRCC were higher for *ABCB4*, *CD36*, *CYP2J2*, *PLIN2*, *ELOVL2*, *APOC1*, *TRIB3*, *LGALS1*, *ENO2*, *MMP1*, *PLA2G2D*, *PIK3R6*, *IL4I1*, *ALOX5*, *PLD4*, and *TNFAIP8L2* compared with those of normal tissues, whereas a lower expression was observed for *ACADSB*, *HSD11B2*, *PTGER3*, *HMGCS2*, *PCK1*, *G6PC*, *ADH6*, *HAO2*, *CYP3A4*, *LPA*, *DPEP1*, *PCK2*, *FABP1*, *REEP6*, *CYP27B1*, *CEL*, *CYP4F3*, and *APOH* (Figures 2C, D). These genes were used for subsequent analysis.

### 3.2 Establishment of a prognostic model

**FIGURE 3:**
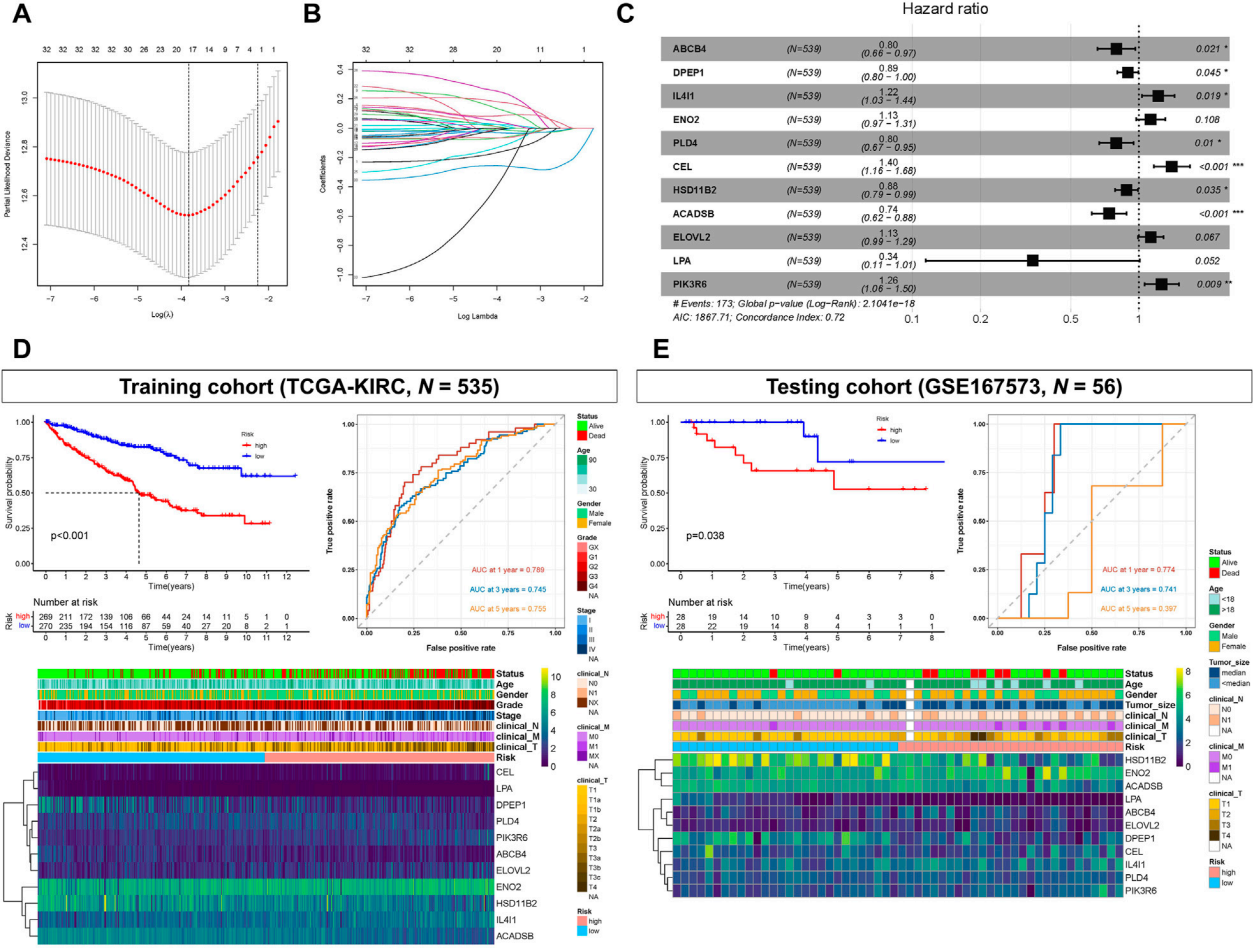
Prognostic model constructed based on lipid metabolism genes (LMGs). **(A, B)** Least absolute shrinkage and selection operator (LASSO) regression complexity (controlled by lambda using the R package “glmnet”). **(C)** Multivariate analysis of risk LMGs in clear cell renal cell carcinoma (ccRCC). **(D)** Survival and receiver operating characteristic (ROC) analyses between high- and low-risk score groups in the training cohort (TCGA-KIRC). **(E)** Survival and ROC analyses between high- and low-risk score groups in the testing cohort (GSE167573).

The 34 OS-related LMGs were included in subsequent LASSO analysis (Figure 3A, B). Following cross validation, 19 genes achieved the minimum partial likelihood deviance (*ABCB4*, *ALOX5*, *DPEP1*, *PTGER3*, *TRIB3*, *IL4I1*, *ENO2*, *G6PC*, *HMGCS2*, *PLIN2*, *PLD4*, *CEL*, *HSD11B2*, *CYP4F3*, *ACADSB*, *MMP1*, *ELOVL2*, *LPA*, and *PIK3R6*). Multivariate Cox regression analysis then established a prognostic model consisting of a risk signature comprising 11 genes (*ABCB4*, *DPEP1*, *IL4I1*, *ENO2*, *PLD4*, *CEL*, *HSD11B2*, *ACADSB*, *ELOVL2*, *LPA*, and *PIK3R6*; Figure 3C). The formula for risk score calculation was as follows: risk score = (−0.228047)*ABCB4 + (−0.110986)*DPEP1 + 0.197628*IL4I1 + 0.120200*ENO2 + (−0.229000)*PLD4 + 0.334393*CEL + (−0.124687)*HSD11B2 + (−0.301579)*ACADSB + 0.121264*ELOVL2 + (−1.078219)*LPA + 0.231621*PIK3R6.

Kaplan-Meier analysis showed that patients with high-risk scores had statistically shorter survival times than those with low-risk scores, both in the training (TCGA-KIRC) and testing cohort (GSE167573) (Figures 3D, E). For ROC analysis, the area under the curve (AUC) for the 1-, 3-, and 5-year survival rates were 0.789, 0.745, and 0.755, respectively, indicating that the model’s predictive effect was good in the training cohort (TCGA-KIRC) (Figure 3D). In the testing cohort (GSE167573), the AUC values for the 1-, 3-, and 5-year survival rates were 0.774, 0.741, and 0.397, respectively (Figure 3E). Additionally, the risk score and these 11 genes were all significantly associated with poor prognoses and histology grades for each of the TCGA-KIRC, GSE167573, and GSE126964 datasets (Figures 3D, E; Supplementary Figure S1).

### 3.3 Gene mutations in different risk groups

**FIGURE 4:**
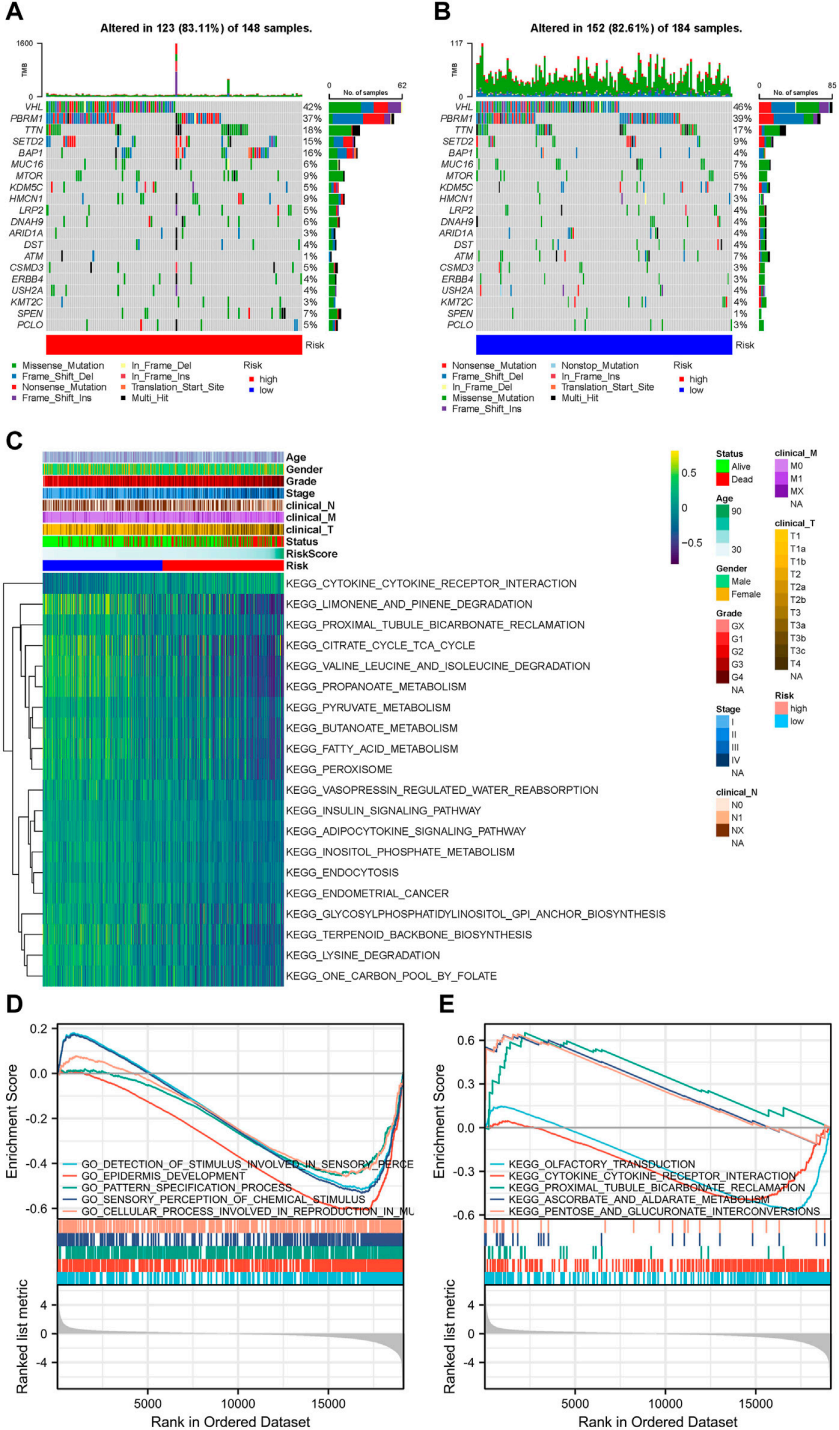
Landscape of mutation profiles and pathway enrichment between patients with high and low-risk clear cell renal cell carcinoma (ccRCC). Waterfall plots representing mutation information in each sample for **(A)** high- and **(B)** low-risk groups. **(C)** Heatmap of KEGG analysis based on risk scores in the TCGA-KIRC dataset. GSEA analysis for **(D)** GO biological process and **(E)** KEGG enrichment in the TCGA-KIRC dataset according to risk score.

According to somatic mutation data, the genes, *VHL*, *PBRM1*, *TTN*, *SETD2*, and *BAP1*, had the highest mutation frequencies. Somatic mutation landscapes of the high- and low-risk groups exhibited a distinct mutation ratio in the TCGA-KIRC cohorts. Most gene mutations were more frequent in the high-risk group than they were in the low-risk group (Figures 4A, B).

### 3.4 Association between ccRCC progression pathways and risk score groups

The GSVA results suggested that LMGs included in the risk score model could modulate pathways of the TCA cycle, adipocytokine signaling, FA metabolism, endometrial cancer, cytokine-cytokine receptor interaction and prostate cancer (Figure 4C). GO biological process and KEGG analyses from GSEA, based on DEGs between the high- and low-risk groups of the TCGA-KIRC dataset, also supported this conclusion (Figures 4D, E).

### 3.5 Immune microenvironment differed between risk groups

**FIGURE 5:**
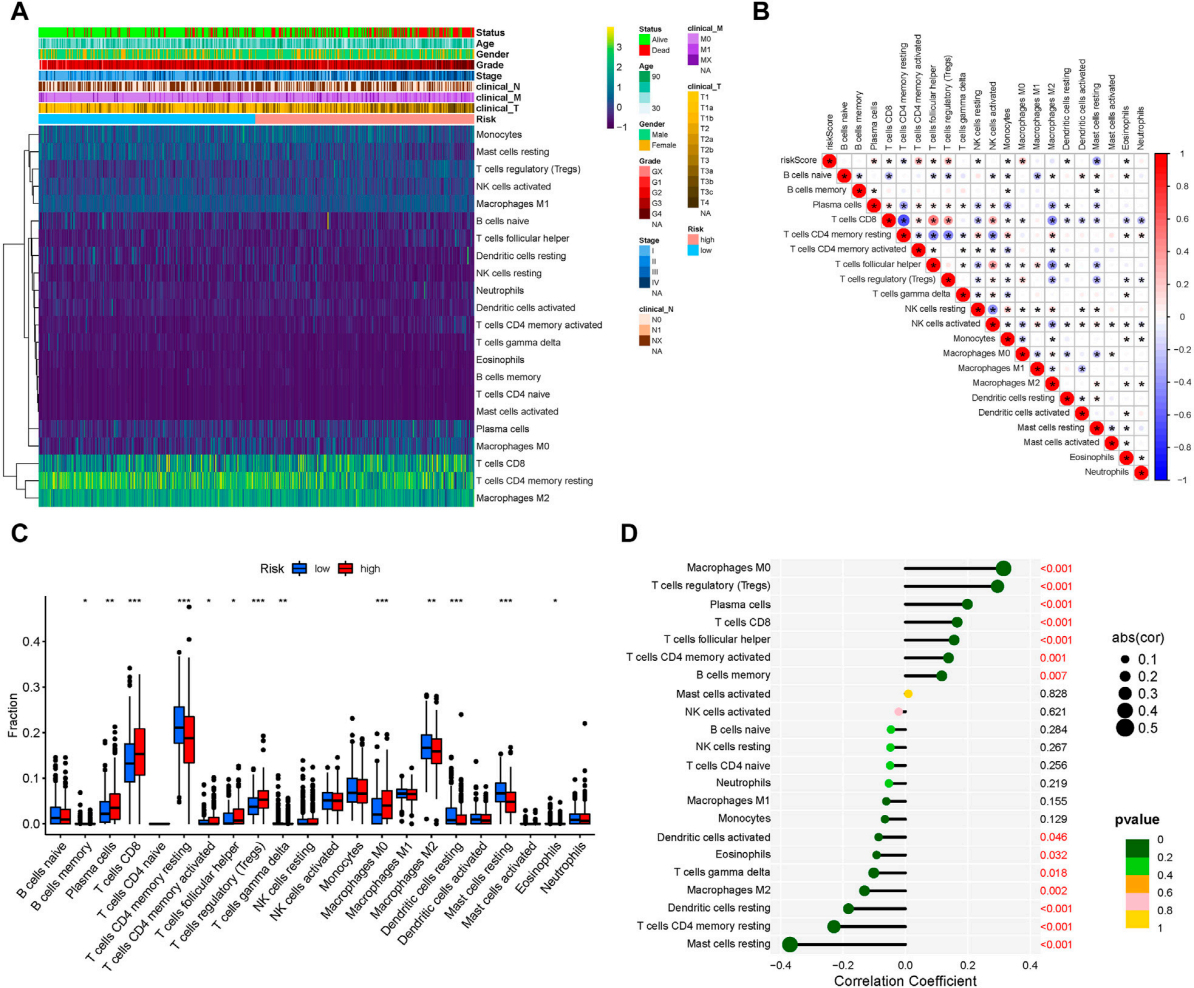
Tumor-infiltrating immune cell landscape estimation. **(A)** Heatmap of 22 infiltrating immune cell types in the TCGA-KIRC dataset. **(B)** Correlation between different infiltrating immune cell subtypes. Blue represents negative correlation and red represents positive correlation. *, *p* < 0.05. **(C)** Differences in distribution of the tumor-infiltrating immune cells between high- and low-risk groups. *p < 0.05, **p < 0.01, ***p < 0.001. **(D)** Correlation analysis of the risk score and different infiltrating immune cell subtypes.

After analyzing tumor-infiltrating immune cell abundance in the TCGA-KIRC dataset using the CIBERSORT algorithm, we drew a heatmap of the 22 infiltrating immune cell types (Figure 5A). Next, we performed correlation analysis between tumor infiltrating immune cells. The highest significantly positive correlation was between activated CD8 and follicular helper T cells, whereas the highest significantly negative correlation was between CD8 and CD4 memory resting T cells (Figure 5B). Comparison analysis revealed that the infiltration level of the “Plasma cells,” “T cells CD8,” “T cells CD4 memory activated,” “T cells follicular helper,” “T cells regulatory (Tregs),” and “Macrophages M0” were significantly higher in the high-risk group than in the low-risk group. However, “B cells memory,” “T cells CD4 memory resting,” “T cells gamma delta,” “Macrophages M2,” “Dendritic cells resting,” “Mast cells resting,” and “Eosinophils” were significantly lower in the high-risk group (Figure 5C). Finally, we analyzed the correlation between infiltrating immune cell types and risk scores. The results showed that Macrophages M0, Tregs, plasma cells, T cells CD8, T cells follicular helper, T cells CD4 memory activated and B cells memory were significantly positively correlated with the risk score, whereas the risk score had a significantly negative correlation with mast cells resting, T cells CD4 memory resting, dendritic cells resting, Macrophages M2, T cells gamma delta, eosinophils and dendritic cells activated (Figure 5D). Collectively, our results show that these 13 cell types (plasma cells, T cells CD8, T cells CD4 memory activated, T cells follicular helper, Tregs, Macrophages M0, Macrophages M2, B cells memory, T cells CD4 memory resting, T cells gamma delta, Dendritic cells resting, mast cells resting, and eosinophils) may play an important role in the lipid metabolism related ccRCC microenvironment.

### 3.6 Single-cell transcriptomic context of prognostic LMGs

**FIGURE 6:**
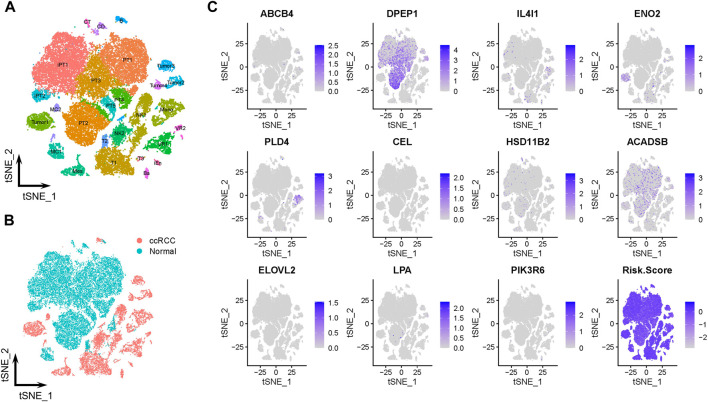
Prognostic expression profile based on single-cell sequencing analysis. **(A, B)** Composition and distribution of single cells from GSE131685 and GSE171306 datasets. **(C)** Expression profiles of *ABCB4*, *DPEP1*, *IL4I1*, *ENO2*, *PLD4*, *CEL*, *HSD11B2*, *ACADSB*, *ELOVL2*, *LPA*, and *PIK3R6*, and risk scores for each cell. t-SNE, t-distributed stochastic neighbor; Im., immune; Epi., epithelial; Endo., endothelial; Mes, mesenchymal; CD, collecting duct; CT, connecting tubule; iEn, injured endothelial cells; Fib, fibroblast; Mast, mast cell; MC, macrophage; Mono, monocyte; PT, proximal tubule; iPT, injured proximal tubule; VR, vasa recta.

The scRNA-Seq data from GSE131685 (containing three healthy kidney samples) and GSE171306 (containing two ccRCC samples) datasets further verified the prognostic model and expression profiles of LMGs. In detail, a total of 27 different cell clusters and five cell groups were identified (Figures 6A, B; Supplementary Figure S2). Calculating the risk scores for each cell and constructing t-SNE and violin plots (Figure 6C; Supplementary Figure S3A, B) revealed that most prognostic LMGs showed differential expression signatures between different cell types (from ccRCC and healthy renal tissues), similarly to the abovementioned results in the transcriptome data.

**FIGURE 7:**
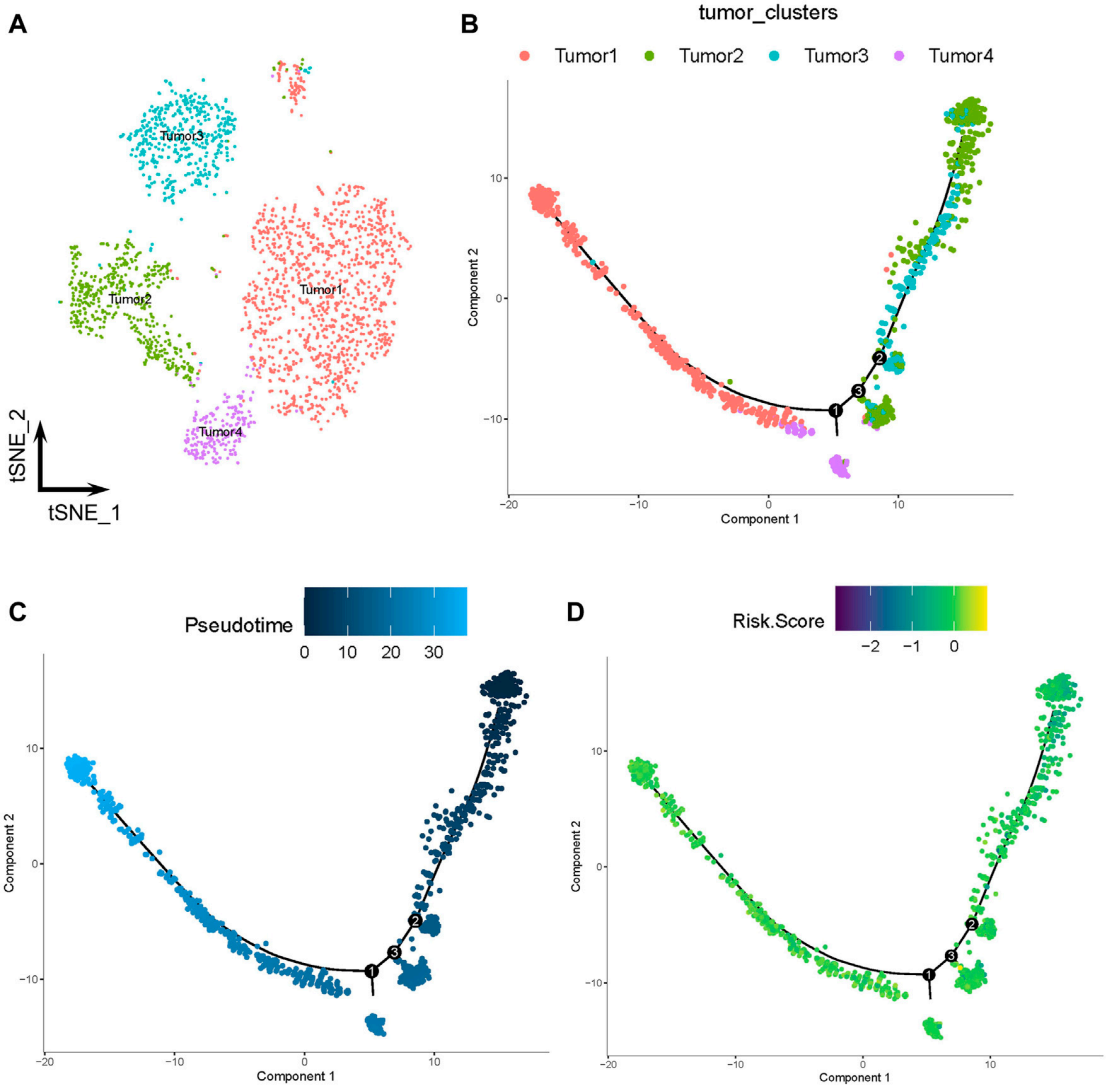
Pseudotime and trajectory analyses of tumor cells. **(A)** The *t*-distributed stochastic neighbor (*t*-SNE) plot of four clusters of tumor cells. **(B, C)** Tendency curve from tumor 2 and tumor 3 clusters to tumor 1 and tumor 4 clusters. The *y*-axis shows the value of principal component 1 (the first principal direction of maximum sample change) and *x*-axis shows the value of principal component 2 (second principal direction of maximum sample change). **(D)** Expression profiles of risk scores annotated in pseudotime and the trajectory plot.

The tumor cells were profiled and arranged into four clusters: tumors 1–4 (Figure 7A). Pseudotime and trajectory analyses revealed a continuous cell fate that started at tumor 2 and tumor 3, then progressed towards tumor 1 (tumor 4 was a transitioning state; Figures 7B, C). The distribution of risk scores was also visualized in relation to LMGs (Figure 7D; Supplementary Figure S4A–C). The risk score results indicated a differentiation trajectory from low- (tumors 2 and 3) to high-risk tumor cells (tumors 4 and 1). Together, these results further validated the predictive effect of the developed LMG prognostic model.

### 3.7 Validation of mRNA and protein expressions of prognostic LMGs

**FIGURE 8:**
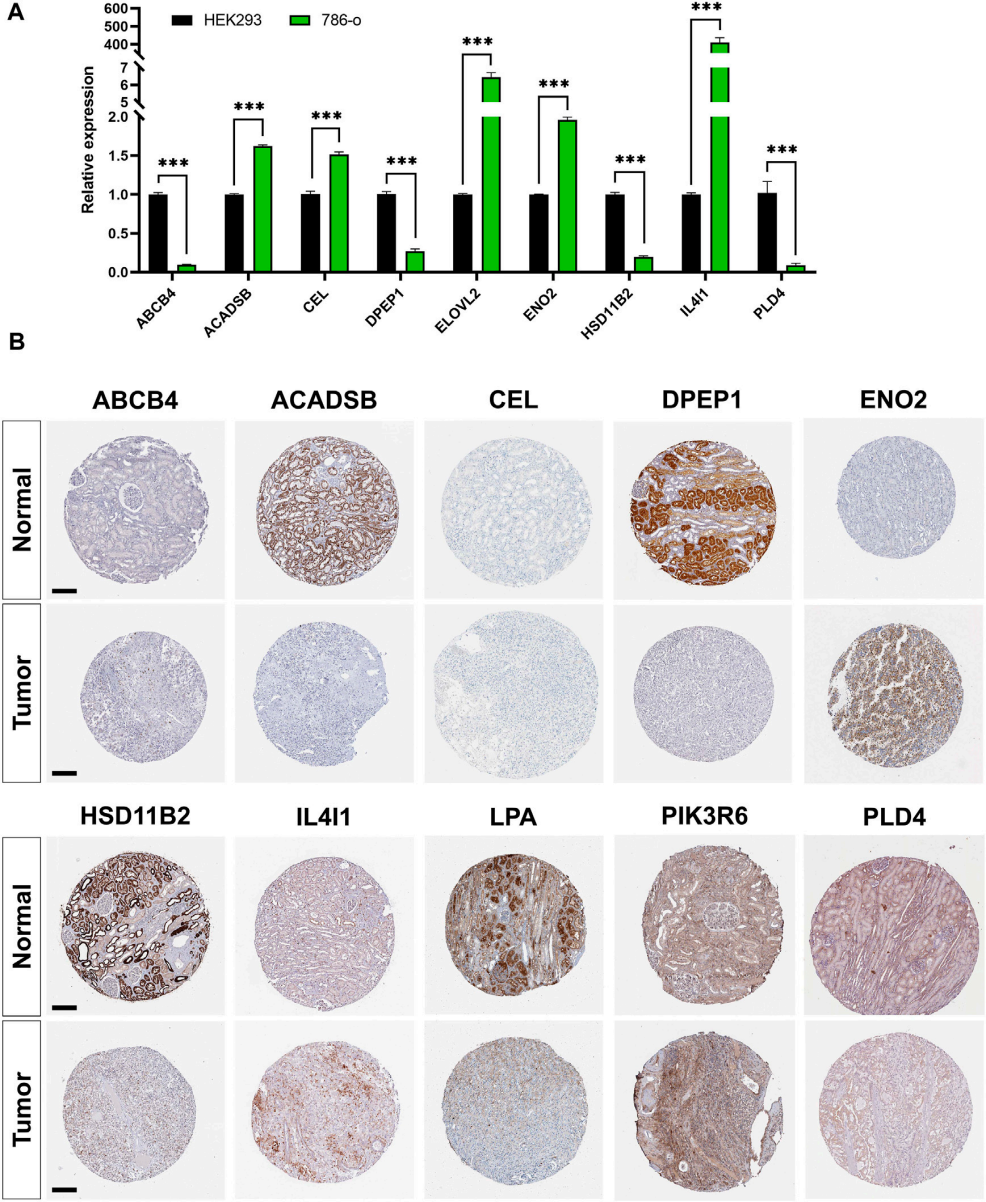
Validation of mRNA and protein expressions of prognostic lipid metabolism genes (LMGs). **(A)** The expression level of *ABCB4*, *DPEP1*, *IL4I1*, *ENO2*, *PLD4*, *CEL*, *HSD11B2*, *ACADSB*, and *ELOVL2* between HEK293 and 786-o cell lines were detected using RT-PCR. Data are shown as mean ± SD. Statistical significance was measured using Student’s *t* test. ^*^ *p* < 0.05; ^**^ *p* < 0.01; ^***^ *p* < 0.001. **(B)** Protein expression levels of ABCB4, DPEP1, IL4I1, ENO2, PLD4, CEL, HSD11B2, ACADSB, LPA, and PIK3R6 in tumor and normal tissues. Bar: 200 μm.

RT-PCR results showed that the expression of *ACADSB*, *CEL*, *ELOVL2*, *ENO2*, and *IL4I1* were significantly higher in the 786-o cell line compared with that in HEK293. However, expression of *ABCB4*, *DPEP1*, *HSD11B2*, and *PLD4* were significantly lower in ccRCC cell lines (Figure 8A). Immunohistochemistry staining results from the HPA database validated the expression levels of prognostic LMGs. ABCB4, ENO2, IL4I1, and PIK3R6 proteins were upregulated in ccRCC samples compared with those in normal controls, whereas expression levels of ACADSB, DPEP1, HSD11B2, and LPA were downregulated (Figure 8B). Collectively, only *ENO2*, *IL4I1*, *DPEP1*, and *HSD11B2* had a similar expression pattern at the transcriptional and translational levels.

## 4 Discussion

The most prevalent type of RCC with a high mortality rate is ccRCC. It is therefore important to identify biomarkers for the early diagnosis of ccRCC. Here, a novel prognostic model was developed based on 11 lipid metabolism genes (*ABCB4*, *DPEP1*, *IL4I1*, *ENO2*, *PLD4*, *CEL*, *HSD11B2*, *ACADSB*, *ELOVL2*, *LPA*, and *PIK3R6*). Eight LMGs (*ABCB4*, *DPEP1*, *IL4I1*, *PLD4*, *CEL*, *HSD11B2*, *ACADSB*, and *PIK3R6*) were also identified as independent prognostic markers for ccRCC *via* integrated bioinformatics analysis.

Metabolic disorders, especially lipid metabolism disorders, are a hallmark for ccRCC progression. The term “clear cell” alludes to the lipid and glycogen-rich cytoplasmic deposits that form in ccRCC tumor cells (Sim and Johnson, 2015). Bianchi et al. (2017) performed oil red staining in ccRCC tissue sections and primary cell cultures, confirming the abundance of lipid-loaded deposits inside tumor cells. However, the detailed mechanism of this lipid storage and its role in ccRCC are unclear. Qiu et al. (2015) proposed that lipid storage in ccRCC cells maintains the integrity of the endoplasmic reticulum (ER) and suppresses cytotoxic ER stress responses, thereby promoting tumor cell survival. At the molecular level, the LoF mutation or downregulation of the von Hippel–Lindau (*VHL*) gene has been identified in over 90% of ccRCC cases (Latif et al., 1993; Noonan et al., 2016). As an E3 ubiquitin ligase, VHL can target alpha subunits of the hypoxia-inducible factor (HIF) heterodimeric transcription factor for ubiquitin-mediated degradation. Following the LoF of VHL, HIFs are constitutively activated (Shen and Kaelin, 2013). Du et al. (2017) recently found that the lipid loading of lipid droplets inside ccRCC cells was driven by repression of carnitine palmitoyltransferase 1A (CPT1A). *CPT1A* is a HIF target gene that participates in the transportation of FA into mitochondria. When HIFs were activated by the downregulation of *VHL*, the system of FA transport entry into the mitochondrion was destroyed by CPT1A suppression, forcing FAs to act as lipid droplets for storage in tumor cells . Here, analysis of ccRCC transcriptome data further confirmed the expression shift of LMGs between ccRCC and normal tissues (Figure 2C, D; Figure 4A, Figure 5, Figure 6C), even in pan-cancer (Supplementary Figure S5). LMGs, including *ABCB4*, *DPEP1*, *IL4I1*, *PLD4*, *CEL*, *HSD11B2*, *ACADSB*, and *PIK3R6*, were also identified as independent prognostic factors for ccRCC.

ATP Binding Cassette (ABC) transporters belong to a family containing various molecules found across extra- and intra-cellular membranes. Many available data have provided evidence for their potential role in cancer development and drug resistance (Fletcher et al., 2010; Nobili et al., 2020). To date, 49 different ABC transporters of seven subfamilies (A–G) have been identified in humans. Among them, ABCA, ABCB, and ABCC are the largest subfamilies. As a member of ABCB, ABCB4 is located in the canalicular membrane of hepatocytes and acts as a translocator of phospholipids into bile, and defects may cause rare biliary diseases (Smit et al., 1993; Wang et al., 2009). Thus, *Abcb4* ^−/−^ mice are widely used as a model for sclerosing cholangitis (Reich et al., 2021). Recently, accumulating evidence indicates that *ABCB4* has a close relationship with tumor progression. Huang et al. (2018) showed that ABCB4 mediated the efflux transport of doxorubicin and contributed to the acquired resistance of the drug in breast cancer cells. Furthermore, *ABCB4* takes part in 5-fluorouracil resistance. Hu et al. (2018) revealed that loss of *ABCB4* may enhance the resistance of colon cancer to 5-fluorouracil *via* inhibiting apoptosis. However, the underlying mechanism of *ABCB4* in ccRCC requires further research.

DPEP1, also known as kidney membrane dipeptidase, is involved in the metabolism of glutathione. DPEP1 is highly expressed in proximal tubular cells and peritubular capillaries of the normal kidney (Choudhury et al., 2019; Nitanai et al., 2002). Lau et al. (2022) reported that *DPEP1* deficiency could block neutrophil adhesion to peritubular capillaries and reduce inflammatory monocyte recruitment to the kidney after ischemia reperfusion injury, and DPEP1 itself could be a potential therapeutic target for acute kidney injury. *DPEP1* has also been implicated in several types of cancers. Ren et al. (2021) identified *DPEP1* as one of six antioxidant genes that regulate ccRCC. Cui X et al. (2019) revealed that the overexpression of *DPEP1* could distinctly activate PI3K/Akt/mTOR signaling, thereby promoting hepatoblastoma cell proliferation, migration, and invasion (Cui X et al., 2019). In our study, *DPEP1* showed significantly low expression in ccRCC (Figures 2C, D). Survival analysis showed that downregulation of *DPEP1* was associated with poor prognosis in ccRCC patients (Figure 3). However, the detailed mechanism underlying the involvement of *DPEP1* in ccRCC remains unclear.

Interleukin-4-induced-1 (IL4I1) is a glycosylated protein that belongs to the l-amino-acid oxidase family (Lasoudris et al., 2011; Molinier-Frenkel et al., 2019). A recent study revealed that *IL4I1* expression was enhanced in most tumor entities compared with that of normal tissues, and IL4I1 is a metabolic immune checkpoint, thereby suppressing adaptive immune responses and promoting chronic lymphocytic leukemia progression (Sadik et al., 2020). Moreover, *IL4I1* was found to play a critical role in the development of ovarian cancer (Zhao et al., 2021), head-neck cancer (Mazzoni et al., 2021), cutaneous melanoma (Prevost-Blondel and Richard, 2019), and ccRCC (Liu et al., 2020). These findings suggest that *IL4I1* might be a potential therapeutic target for patients with ccRCC.

Phospholipase D4 (PLD4) is a member of the phospholipid enzyme family. Previous research has found that the expression of PLD4 is upregulated in mice and human kidneys after fibrosis. Blocking PLD4 expression protected mice from folic acid–induced kidney fibrosis and inhibited the increase in TGF-β signaling (Trivedi et al., 2017). However, the potential roles of *PLD4* in tumor progression is still largely unknown. Only Gao et al. (2017) reported that *PLD4* might promote the activation of M1 macrophages and thereby suppress colon cancer. Here, we firstly identified *PLD4* as an independent prognostic marker for ccRCC, though in-depth research into its role in ccRCC is still lacking.

Carboxyl ester lipase (CEL) is a lipolytic enzyme that can hydrolyze a wide variety of lipid substrates, including cholesteryl esters, glycerides, phospholipids, and ceramide (Hui et al., 2002). CEL is mainly expressed in the pancreas and lactating mammary glands (Xiao et al., 2016). As a novel tumor-associated gene, *CEL* has been implicated in breast (Cui Y et al., 2019) and pancreatic cancers (Dalva et al., 2017). However, the underlying mechanism by which *CEL* participates in tumor progression requires further research.

In the TME, the above 11 genes (*ABCB4*, *DPEP1*, *IL4I1*, *ENO2*, *PLD4*, *CEL*, *HSD11B2*, *ACADSB*, *ELOVL2*, *LPA*, and *PIK3R6*) related to lipid metabolism can also act on immune cells and even affect immunotherapy. In head and neck squamous cell carcinoma, *IL4I1* was confirmed to inhibit T cell proliferation (Mazzoni et al., 2021). The aryl hydrocarbon receptor (AHR) can enhance tumor malignancy and inhibit antitumor immunity. IL4I1 was found to be strongly associated with AHR activity in 32 solid tumors. The combination of immune checkpoint blockade (ICB) and IL4I1 inhibitors is expected to play a therapeutic role in solid tumors such as renal cancer and glioma (Sadik et al., 2020). The study indicated that *DPEP1* can be used as an independent predictor of prognosis in patients with RCC, and is expected to be used as a target of immunotherapy, providing a new avenue for the immunotherapy of renal cancer (Ren et al., 2021). *PIK3R6* is used in combination with ICB (atezolizumab) as one of the tumor vaccine antigens. In the treatment of metastatic castration-resistant prostate cancer, a Phase Ib clinical trial demonstrated that the combination treatment is safe, well tolerated and beneficial to patients (Dorff et al., 2021).

Our study had some limitations. First, we screened DEGs from kidney and para-cancer tissues in TCGA (TCGA-KIRC) and GEO (GSE126964) databases. However, the sample size of cancer tissues was much larger than that of para-cancer tissues and, might have resulted in statistical errors for the screened DEGs. Second, the dataset for verification, GSE167573, lacked sufficient clinical data and, could not be verified and analyzed using TNM staging and other clinical data using our model. Third, further clinical cases and tissue specimens need to be collected to verify the clinical effectiveness and reliability of our model.

## 5 Conclusion

In summary, LMG expression was shown to be associated with the survival outcomes of patients with ccRCC. A novel risk score model based on a signature of 11 LMGs (*ABCB4*, *DPEP1*, *IL4I1*, *ENO2*, *PLD4*, *CEL*, *HSD11B2*, *ACADSB*, *ELOVL2*, *LPA*, and *PIK3R6*) was established. This model was shown to be capable of predicting survival outcomes. Furthermore, LMGs were identified to have the potential to become therapeutic targets for ccRCC.

## Data Availability

The datasets presented in this study can be found in online repositories. The names of the repository/repositories and accession number(s) can be found in the article/Supplementary Material.

## References

[B1] Atkins M. B. Tannir N. M. (2018). Current and emerging therapies for first-line treatment of metastatic clear cell renal cell carcinoma. Cancer Treat. Rev. 70, 127–137. 10.1016/j.ctrv.2018.07.009 30173085

[B2] Bianchi C. Meregalli C. Bombelli S. Di Stefano V. Salerno F. Torsello B. (2017). The glucose and lipid metabolism reprogramming is grade-dependent in clear cell renal cell carcinoma primary cultures and is targetable to modulate cell viability and proliferation. Oncotarget 8 (69), 113502–113515. 10.18632/oncotarget.23056 29371925 PMC5768342

[B3] Chakraborty S. Balan M. Sabarwal A. Choueiri T. K. Pal S. (2021). Metabolic reprogramming in renal cancer: Events of a metabolic disease. Biochim. Biophys. Acta-Rev. Cancer. 1876 (1), 188559. 10.1016/j.bbcan.2021.188559 33965513 PMC8349779

[B4] Choudhury S. R. Babes L. Rahn J. J. Ahn B. Y. Goring K. R. King J. C. (2019). Dipeptidase-1 is an adhesion receptor for neutrophil recruitment in lungs and liver. Cell. 178 (5), 1205–1221. 10.1016/j.cell.2019.07.017 31442408

[B5] Cui X X. Liu X. Han Q. Zhu J. Li J. Ren Z. (2019). DPEP1 is a direct target of miR-193a-5p and promotes hepatoblastoma progression by PI3K/Akt/mTOR pathway. Cell. Death Dis. 10 (10), 701. 10.1038/s41419-019-1943-0 31541079 PMC6754441

[B6] Cui Y Y. Jiao Y. Wang K. He M. Yang Z. (2019). A new prognostic factor of breast cancer: High carboxyl ester lipase expression related to poor survival. Cancer Genet. 239, 54–61. 10.1016/j.cancergen.2019.09.005 31561066

[B7] Dalva M. El J. K. Steine S. J. Johansson B. B. Ringdal M. Torsvik J. (2017). Copy number variants and VNTR length polymorphisms of the carboxyl-ester lipase (CEL) gene as risk factors in pancreatic cancer. Pancreatology 17 (1), 83–88. 10.1016/j.pan.2016.10.006 27773618

[B8] Dorff T. Hirasawa Y. Acoba J. Pagano I. Tamura D. Pal S. (2021). Phase Ib study of patients with metastatic castrate-resistant prostate cancer treated with different sequencing regimens of atezolizumab and sipuleucel-T. J. Immunother. Cancer. 9 (8), e002931. 10.1136/jitc-2021-002931 34376554 PMC8356194

[B9] Du W. Zhang L. Brett-Morris A. Aguila B. Kerner J. Hoppel C. L. (2017). HIF drives lipid deposition and cancer in ccRCC via repression of fatty acid metabolism. Nat. Commun. 8 (1), 1769. 10.1038/s41467-017-01965-8 29176561 PMC5701259

[B10] Ferro M. de Cobelli O. Musi G. Del G. F. Carrieri G. Busetto G. M. (2022). Radiomics in prostate cancer: An up-to-date review. Ther. Adv. Urol. 14, 17562872221109020. 10.1177/17562872221109020 35814914 PMC9260602

[B11] Fletcher J. I. Haber M. Henderson M. J. Norris M. D. (2010). ABC transporters in cancer: More than just drug efflux pumps. Nat. Rev. Cancer. 10 (2), 147–156. 10.1038/nrc2789 20075923

[B12] Gao L. Zhou Y. Zhou S. X. Yu X. J. Xu J. M. Zuo L. (2017). PLD4 promotes M1 macrophages to perform antitumor effects in colon cancer cells. Oncol. Rep. 37 (1), 408–416. 10.3892/or.2016.5216 27840999

[B13] Hanzelmann S. Castelo R. Guinney J. (2013). Gsva: Gene set variation analysis for microarray and rna-seq data. BMC Bioinforma. 14, 7. 10.1186/1471-2105-14-7 PMC3618321 23323831

[B14] Hao Y. Hao S. Andersen-Nissen E. Mauck W. R. Zheng S. Butler A. (2021). Integrated analysis of multimodal single-cell data. Cell. 184 (13), 3573–3587.e29. 10.1016/j.cell.2021.04.048 34062119 PMC8238499

[B15] Horiguchi A. Asano T. Asano T. Ito K. Sumitomo M. Hayakawa M. (2008). Pharmacological inhibitor of fatty acid synthase suppresses growth and invasiveness of renal cancer cells. J. Urol. 180 (2), 729–736. 10.1016/j.juro.2008.03.186 18555493

[B16] Hsieh J. J. Purdue M. P. Signoretti S. Swanton C. Albiges L. Schmidinger M. (2017). Renal cell carcinoma. Nat. Rev. Dis. Prim. 3, 17009. 10.1038/nrdp.2017.9 28276433 PMC5936048

[B17] Hu H. Wang M. Guan X. Yuan Z. Liu Z. Zou C. (2018). Loss of ABCB4 attenuates the caspase-dependent apoptosis regulating resistance to 5-Fu in colorectal cancer. Biosci. Rep. 38 (1). 10.1042/BSR20171428 PMC5821943 29371412

[B18] Huang J. F. Wen C. J. Zhao G. Z. Dai Y. Li Y. Wu L. X. (2018). Overexpression of abcb4 contributes to acquired doxorubicin resistance in breast cancer cells *in vitro* . Cancer Chemother. Pharmacol. 82 (2), 199–210. 10.1007/s00280-018-3603-y 29777275

[B19] Huang H. Zhu L. Huang C. Dong Y. Fan L. Tao L. (2021). Identification of hub genes associated with clear cell renal cell carcinoma by integrated bioinformatics analysis. Front. Oncol. 11, 726655. 10.3389/fonc.2021.726655 34660292 PMC8516333

[B20] Hui D. Y. Howles P. N. (2002). Carboxyl ester lipase: Structure-function relationship and physiological role in lipoprotein metabolism and atherosclerosis. J. Lipid Res. 43 (12), 2017–2030. 10.1194/jlr.r200013-jlr200 12454261

[B21] Humphrey P. A. Moch H. Cubilla A. L. Ulbright T. M. Reuter V. E. (2016). The 2016 WHO classification of tumours of the urinary system and male genital organs-Part B: Prostate and bladder tumours. Eur. Urol. 70 (1), 106–119. 10.1016/j.eururo.2016.02.028 26996659

[B22] Kim Y. S. Jung J. Jeong H. Lee J. H. Oh H. E. Lee E. S. (2019). High membranous expression of fatty acid transport protein 4 is associated with tumorigenesis and tumor progression in clear cell renal cell carcinoma. J. Artic. Dis. Markers. 2019, 5702026. 10.1155/2019/5702026 PMC6476224 31089396

[B23] Lasoudris F. Cousin C. Prevost-Blondel A. Martin-Garcia N. Abd-Alsamad I. Ortonne N. (2011). IL4I1: An inhibitor of the CD8⁺ antitumor T-cell response *in vivo* . Eur. J. Immunol. 41 (6), 1629–1638. 10.1002/eji.201041119 21469114 PMC3472400

[B24] Latif F. Tory K. Gnarra J. Yao M. Duh F. M. Orcutt M. L. (1993). Identification of the von Hippel-Lindau disease tumor suppressor gene. Science 260 (5112), 1317–1320. 10.1126/science.8493574 8493574

[B25] Lau A. Rahn J. J. Chappellaz M. Chung H. Benediktsson H. Bihan D. (2022). Dipeptidase-1 governs renal inflammation during ischemia reperfusion injury. Sci. Adv. 8 (5), eabm0142. 10.1126/sciadv.abm0142 35108057 PMC8809686

[B26] Li H. Y. Appelbaum F. R. Willman C. L. Zager R. A. Banker D. E. (2003). Cholesterol-modulating agents kill acute myeloid leukemia cells and sensitize them to therapeutics by blocking adaptive cholesterol responses. Blood 101 (9), 3628–3634. 10.1182/blood-2002-07-2283 12506040

[B27] Li J. Li Q. Su Z. Sun Q. Zhao Y. Feng T. (2020). Lipid metabolism gene-wide profile and survival signature of lung adenocarcinoma. Lipids Health Dis. 19 (1), 222. 10.1186/s12944-020-01390-9 33050938 PMC7557101

[B28] Liao J. Yu Z. Chen Y. Bao M. Zou C. Zhang H. (2020). Single-cell RNA sequencing of human kidney. Sci. Data. 7 (1), 4. 10.1038/s41597-019-0351-8 31896769 PMC6940381

[B29] Liu M. Pan Q. Xiao R. Yu Y. Lu W. Wang L. (2020). A cluster of metabolism-related genes predict prognosis and progression of clear cell renal cell carcinoma. Sci. Rep. 10 (1), 12949. 10.1038/s41598-020-67760-6 32737333 PMC7395775

[B30] Mazzoni A. Capone M. Ramazzotti M. Vanni A. Locatello L. G. Gallo O. (2021). IL4I1 is expressed by head-neck cancer-derived mesenchymal stromal cells and contributes to suppress T cell proliferation. J. Clin. Med. 10 (10), 2111. 10.3390/jcm10102111 34068423 PMC8153554

[B31] Molinier-Frenkel V. Prevost-Blondel A. Castellano F. (2019). The il4i1 enzyme: A new player in the immunosuppressive tumor microenvironment. Cells 8 (7). 10.3390/cells8070757 PMC6678094 31330829

[B32] Motzer R. J. Banchereau R. Hamidi H. Powles T. McDermott D. Atkins M. B. (2020). Molecular subsets in renal cancer determine outcome to checkpoint and angiogenesis blockade. Cancer Cell. 38 (6), 803–817.e4. 10.1016/j.ccell.2020.10.011 33157048 PMC8436590

[B33] Nagao K. Shinohara N. Smit F. de Weijert M. Jannink S. Owada Y. (2018). Fatty acid binding protein 7 may be a marker and therapeutic targets in clear cell renal cell carcinoma. BMC Cancer 18 (1), 1114. 10.1186/s12885-018-5060-8 30442117 PMC6238291

[B34] Newman A. M. Liu C. L. Green M. R. Gentles A. J. Feng W. Xu Y. (2015). Robust enumeration of cell subsets from tissue expression profiles. Nat. Methods. 12 (5), 453–457. 10.1038/nmeth.3337 25822800 PMC4739640

[B35] Nitanai Y. Satow Y. Adachi H. Tsujimoto M. (2002). Crystal structure of human renal dipeptidase involved in beta-lactam hydrolysis. J. Mol. Biol. 321 (2), 177–184. 10.1016/s0022-2836(02)00632-0 12144777

[B36] Nobili S. Lapucci A. Landini I. Coronnello M. Roviello G. Mini E. (2020). Role of atp-binding cassette transporters in cancer initiation and progression. Semin. Cancer Biol. 60, 72–95. 10.1016/j.semcancer.2019.08.006 31412294

[B37] Noonan H. R. Metelo A. M. Kamei C. N. Peterson R. T. Drummond I. A. Iliopoulos O. (2016). Loss of vhl in the zebrafish pronephros recapitulates early stages of human clear cell renal cell carcinoma. Dis. Model. Mech. 9 (8), 873–884. 10.1242/dmm.024380 27491085 PMC5007981

[B38] Obradovic A. Chowdhury N. Haake S. M. Ager C. Wang V. Vlahos L. (2021). Single-cell protein activity analysis identifies recurrence-associated renal tumor macrophages. Cell. 184 (11), 2988–3005.e16. 10.1016/j.cell.2021.04.038 34019793 PMC8479759

[B39] Prevost-Blondel A. Richard Y. (2019). Interleukin 4-induced gene 1 as an emerging regulator of B-cell Biology and its role in cutaneous melanoma. Crit. Rev. Immunol. 39 (1), 39–57. 10.1615/CritRevImmunol.2019030020 31679193

[B40] Qiu B. Ackerman D. Sanchez D. J. Li B. Ochocki J. D. Grazioli A. (2015). Hif2alpha-dependent lipid storage promotes endoplasmic reticulum homeostasis in clear-cell renal cell carcinoma. Cancer Discov. 5 (6), 652–667. 10.1158/2159-8290.CD-14-1507 25829424 PMC4456212

[B41] Qiu X. Hill A. Packer J. Lin D. Ma Y. A. Trapnell C. (2017). Single-cell mrna quantification and differential analysis with census. Nat. Methods. 14 (3), 309–315. 10.1038/nmeth.4150 28114287 PMC5330805

[B42] Qu Y. Y. Zhao R. Zhang H. L. Zhou Q. Xu F. J. Zhang X. (2020). Inactivation of the AMPK-GATA3-ECHS1 pathway induces fatty acid synthesis that promotes clear cell renal cell carcinoma growth. Cancer Res. 80 (2), 319–333. 10.1158/0008-5472.CAN-19-1023 31690668

[B43] Reich M. Spomer L. Klindt C. Fuchs K. Stindt J. Deutschmann K. (2021). Downregulation of TGR5 (GPBAR1) in biliary epithelial cells contributes to the pathogenesis of sclerosing cholangitis. J. Hepatol. 75 (3), 634–646. 10.1016/j.jhep.2021.03.029 33872692

[B44] Ren X. Ma L. Wang N. Zhou R. Wu J. Xie X. (2021). Antioxidant gene signature impacts the immune infiltration and predicts the prognosis of kidney renal clear cell carcinoma. Front. Genet. 12, 721252. 10.3389/fgene.2021.721252 34490047 PMC8416991

[B45] Ritchie M. E. Phipson B. Wu D. Hu Y. Law C. W. Shi W. (2015). Limma powers differential expression analyses for RNA-sequencing and microarray studies. Nucleic Acids Res. 43 (7), e47. 10.1093/nar/gkv007 25605792 PMC4402510

[B46] Sadik A. Somarribas P. L. Ozturk S. Mohapatra S. R. Panitz V. Secker P. F. (2020). IL4I1 is a metabolic immune checkpoint that activates the AHR and promotes tumor progression. Cell. 182 (5), 1252–1270. 10.1016/j.cell.2020.07.038 32818467

[B47] Sanchez-Gastaldo A. Kempf E. Gonzalez D. A. A. Duran I. (2017). Systemic treatment of renal cell cancer: A comprehensive review. Cancer Treat. Rev. 60, 77–89. 10.1016/j.ctrv.2017.08.010 28898679

[B48] Shen C. Kaelin W. J. (2013). The VHL/HIF axis in clear cell renal carcinoma. Semin. Cancer Biol. 23 (1), 18–25. 10.1016/j.semcancer.2012.06.001 22705278 PMC3663044

[B49] Shen D. Gao Y. Huang Q. Xuan Y. Yao Y. Gu L. (2021). E2f1 promotes proliferation and metastasis of clear cell renal cell carcinoma via activation of srebp1-dependent fatty acid biosynthesis. Cancer Lett. 514, 48–62. 10.1016/j.canlet.2021.05.012 34019961

[B50] Siegel R. L. Miller K. D. Fuchs H. E. Jemal A. (2022). Cancer statistics, 2022. CA-Cancer J. Clin. 72 (1), 7–33. 10.3322/caac.21708 35020204

[B51] Sim J. Johnson R. S. (2015). Through a clear cell, darkly: HIF2α/PLIN2-Maintained fat droplets protect ccRCCs from ER stress. Cancer Discov. 5 (6), 584–585. 10.1158/2159-8290.CD-15-0480 26037916

[B52] Smit J. J. Schinkel A. H. Oude E. R. Groen A. K. Wagenaar E. van Deemter L. (1993). Homozygous disruption of the murine mdr2 P-glycoprotein gene leads to a complete absence of phospholipid from bile and to liver disease. Cell. 75 (3), 451–462. 10.1016/0092-8674(93)90380-9 8106172

[B53] Sun G. Chen J. Liang J. Yin X. Zhang M. Yao J. (2021). Integrated exome and RNA sequencing of TFE3-translocation renal cell carcinoma. Nat. Commun. 12 (1), 5262. 10.1038/s41467-021-25618-z 34489456 PMC8421377

[B54] Sung H. Ferlay J. Siegel R. L. Laversanne M. Soerjomataram I. Jemal A. (2021). Global cancer statistics 2020: GLOBOCAN estimates of incidence and mortality worldwide for 36 cancers in 185 countries. CA-Cancer J. Clin. 71 (3), 209–249. 10.3322/caac.21660 33538338

[B55] Trivedi P. Kumar R. K. Iyer A. Boswell S. Gerarduzzi C. Dadhania V. P. (2017). Targeting phospholipase D4 attenuates kidney fibrosis. J. Am. Soc. Nephrol. 28 (12), 3579–3589. 10.1681/ASN.2016111222 28814511 PMC5698063

[B56] Wang D. Q. Cohen D. E. Carey M. C. (2009). Biliary lipids and cholesterol gallstone disease. J. Lipid Res. 50, S406–S411. 10.1194/jlr.R800075-JLR200 19017613 PMC2674701

[B57] Xia L. Oyang L. Lin J. Tan S. Han Y. Wu N. (2021). The cancer metabolic reprogramming and immune response. Mol. Cancer 20 (1), 28. 10.1186/s12943-021-01316-8 33546704 PMC7863491

[B58] Xiao X. Jones G. Sevilla W. A. Stolz D. B. Magee K. E. Haughney M. (2016). A carboxyl ester lipase (CEL) mutant causes chronic pancreatitis by forming intracellular aggregates that activate apoptosis. J. Biol. Chem. 291 (44), 23224–23236. 10.1074/jbc.M116.734384 27650499 PMC5087739

[B59] Yu W. Lei Q. Yang L. Qin G. Liu S. Wang D. (2021). Contradictory roles of lipid metabolism in immune response within the tumor microenvironment. J. Hematol. Oncol. 14 (1), 187. 10.1186/s13045-021-01200-4 34742349 PMC8572421

[B60] Yu Z. Lu W. Su C. Lv Y. Ye Y. Guo B. (2021). Single-cell rna-seq identification of the cellular molecular characteristics of sporadic bilateral clear cell renal cell carcinoma. Front. Oncol. 11, 659251. 10.3389/fonc.2021.659251 34168986 PMC8217644

[B61] Zhao Q. Xue J. Hong B. Qian W. Liu T. Fan B. (2020). Transcriptomic characterization and innovative molecular classification of clear cell renal cell carcinoma in the Chinese population. Cancer Cell. Int. 20, 461. 10.1186/s12935-020-01552-w 32982583 PMC7510315

[B62] Zhao H. Teng Y. Hao W. Li J. Li Z. Chen Q. (2021). Single-cell analysis revealed that IL4I1 promoted ovarian cancer progression. J. Transl. Med. 19 (1), 454. 10.1186/s12967-021-03123-7 34717685 PMC8557560

