# Supplementary material for: A novel lipid metabolism gene signature for clear cell renal cell carcinoma using integrated bioinformatics analysis
Source: Front Cell Dev Biol. 2023 Feb 14;11:1078759. doi: 10.3389/fcell.2023.1078759 (PMC9971983; doi:10.3389/fcell.2023.1078759)
Supplement: Supplementary file 1 [file DataSheet1.docx]

Supplementary Figures


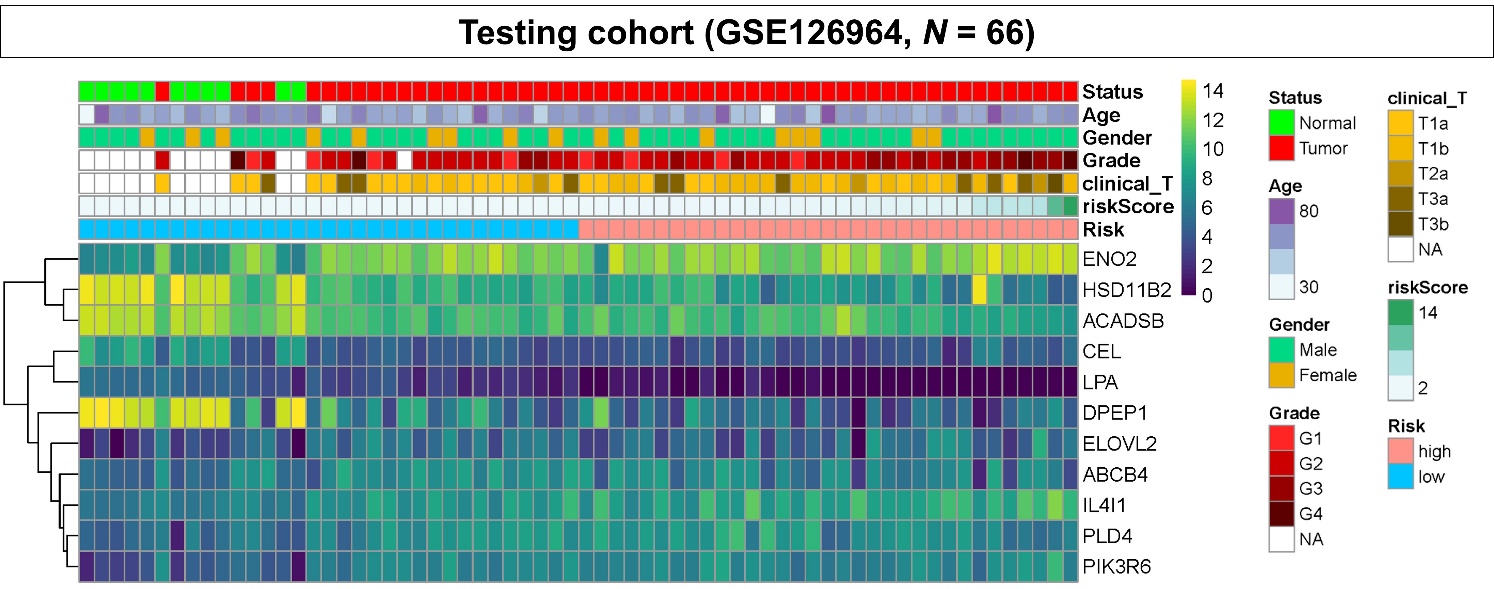


**Supplementary** **Figure 1.** Survival and ROC analyses between high- and low-risk score groups in the another testing cohort (GSE126964).


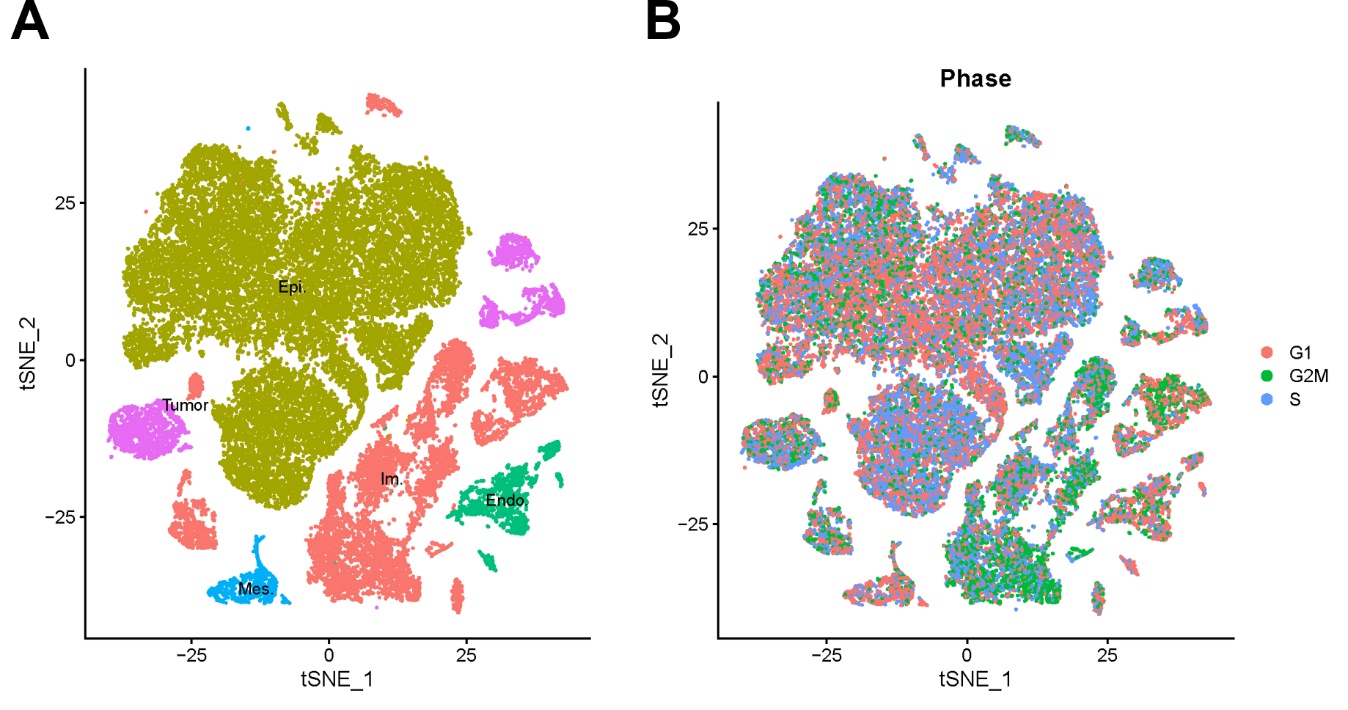


**Supplementary** **Figure 2.** T-SNE plot of **(A)** composition and **(B)** cell cycle phase of single cells from GSE131685 and GSE171306 databases. t-SNE, t-distributed stochastic neighbor; Im., immune; Epi., epithelial; Endo., endothelial; Mes, mesenchymal.


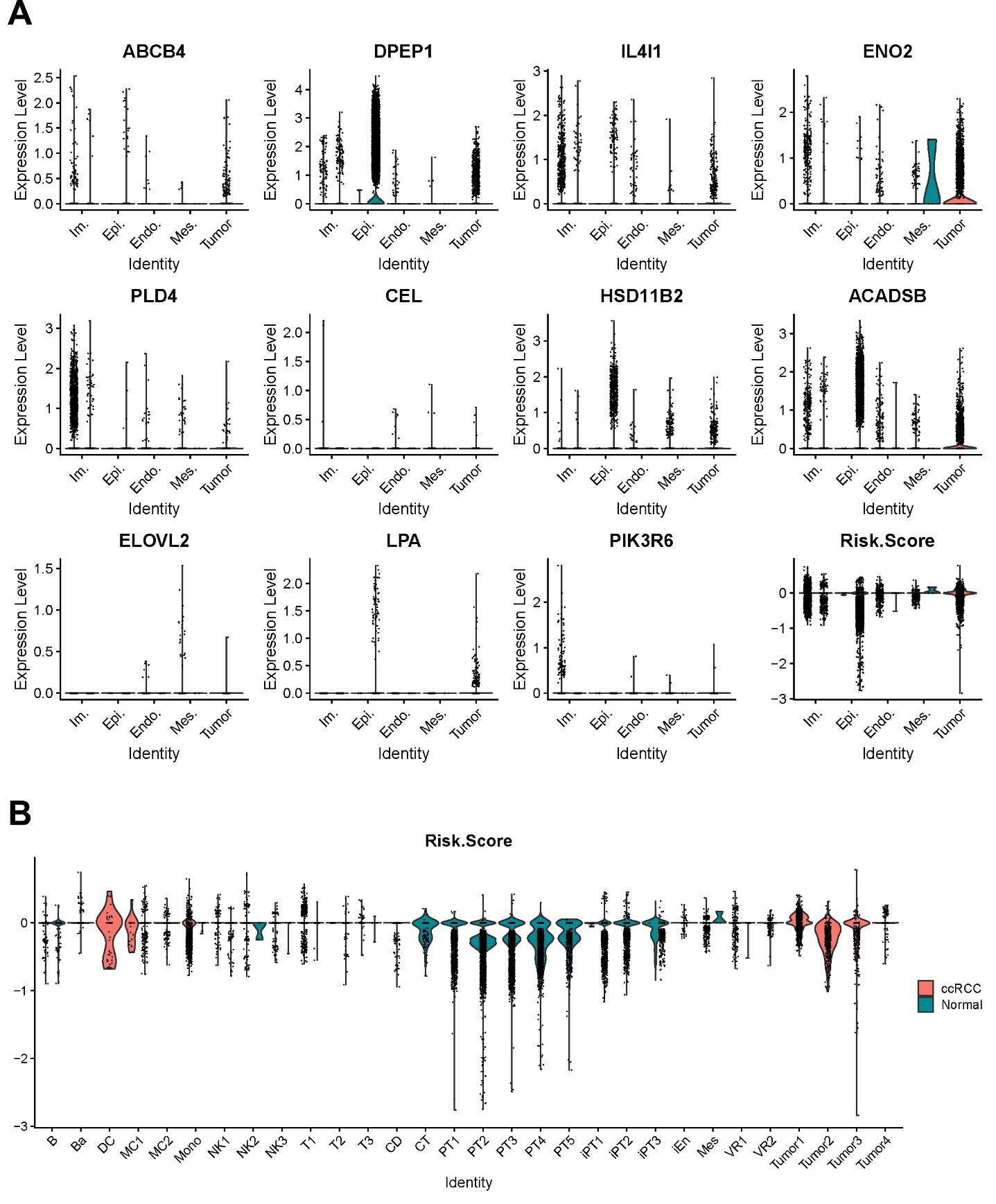


**Supplementary** **Figure 3. (A)** Violin plots of expression profiles of *ABCB4*, *DPEP1*, *IL4I1*, *ENO2*, *PLD4*, *CEL*, *HSD11B2*, *ACADSB*, *ELOVL2*, *LPA*, *PIK3R6*, and risk scores for each cell groups (immune, epithelial, endothelial, mesenchymal, and tumor cells). **(B)** Violin plot of expression profile of risk scores for each cell clusters. ccRCC, clear cell renal cell carcinoma; Im., immune; Epi., epithelial; Endo., endothelial; Mes, mesenchymal; CD, collecting duct; CT, connecting tubule; iEn, injured endothelial cells; Fib, fibroblast; Mast, mast cell; MC, macrophage; Mono, monocyte; PT, proximal tubule; iPT, injured proximal tubule; VR, vasa recta.


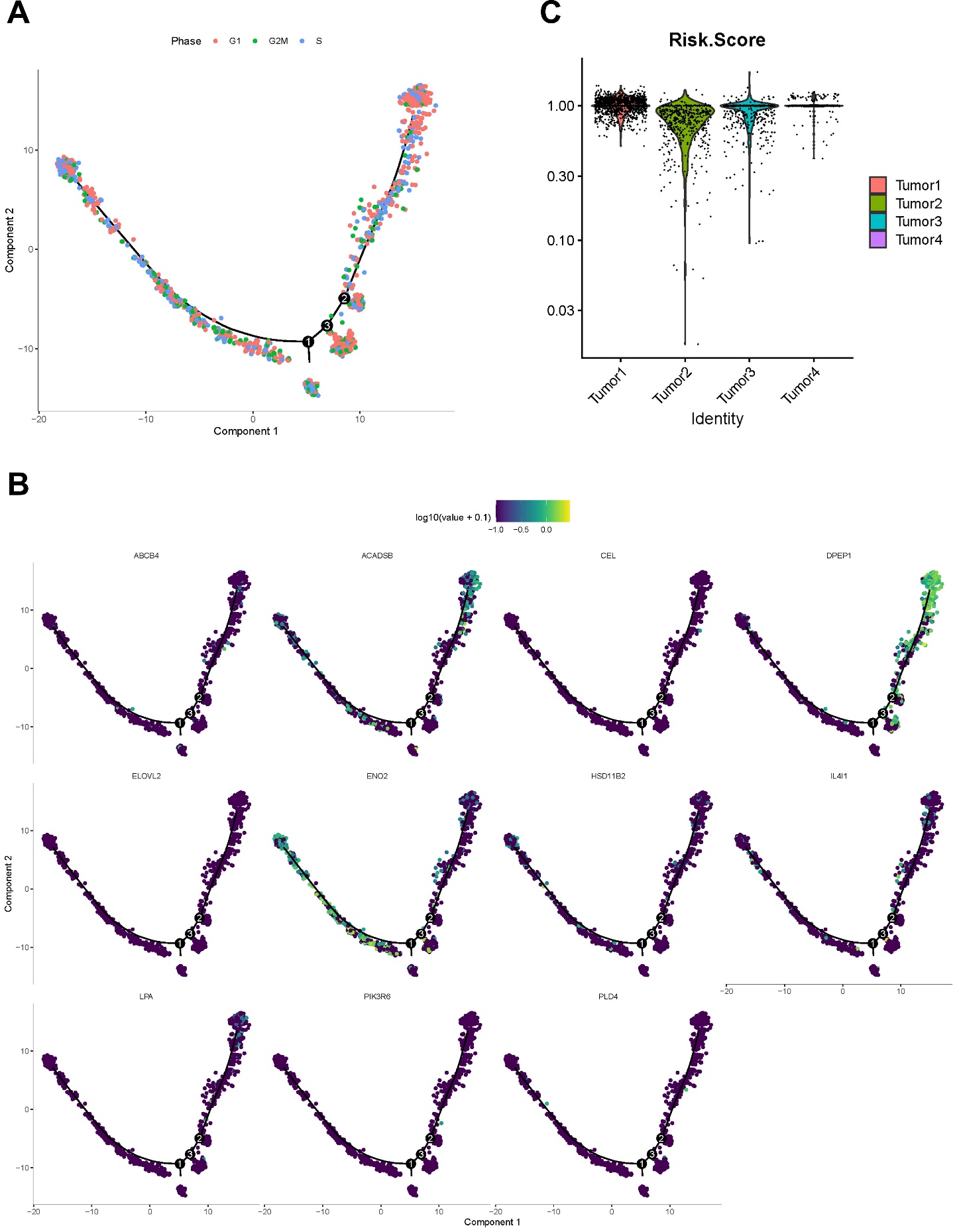


**Supplementary** **Figure 4.** Expression profiles of **(A)** cell cycle phase and **(B)** *ABCB4*, *DPEP1*, *IL4I1*, *ENO2*, *PLD4*, *CEL*, *HSD11B2*, *ACADSB*, *ELOVL2*, *LPA*, and *PIK3R6* annotated in pseudotime and trajectory plot. **(C)** Violin plot of expression profile of risk scores for tumor cell cluster.


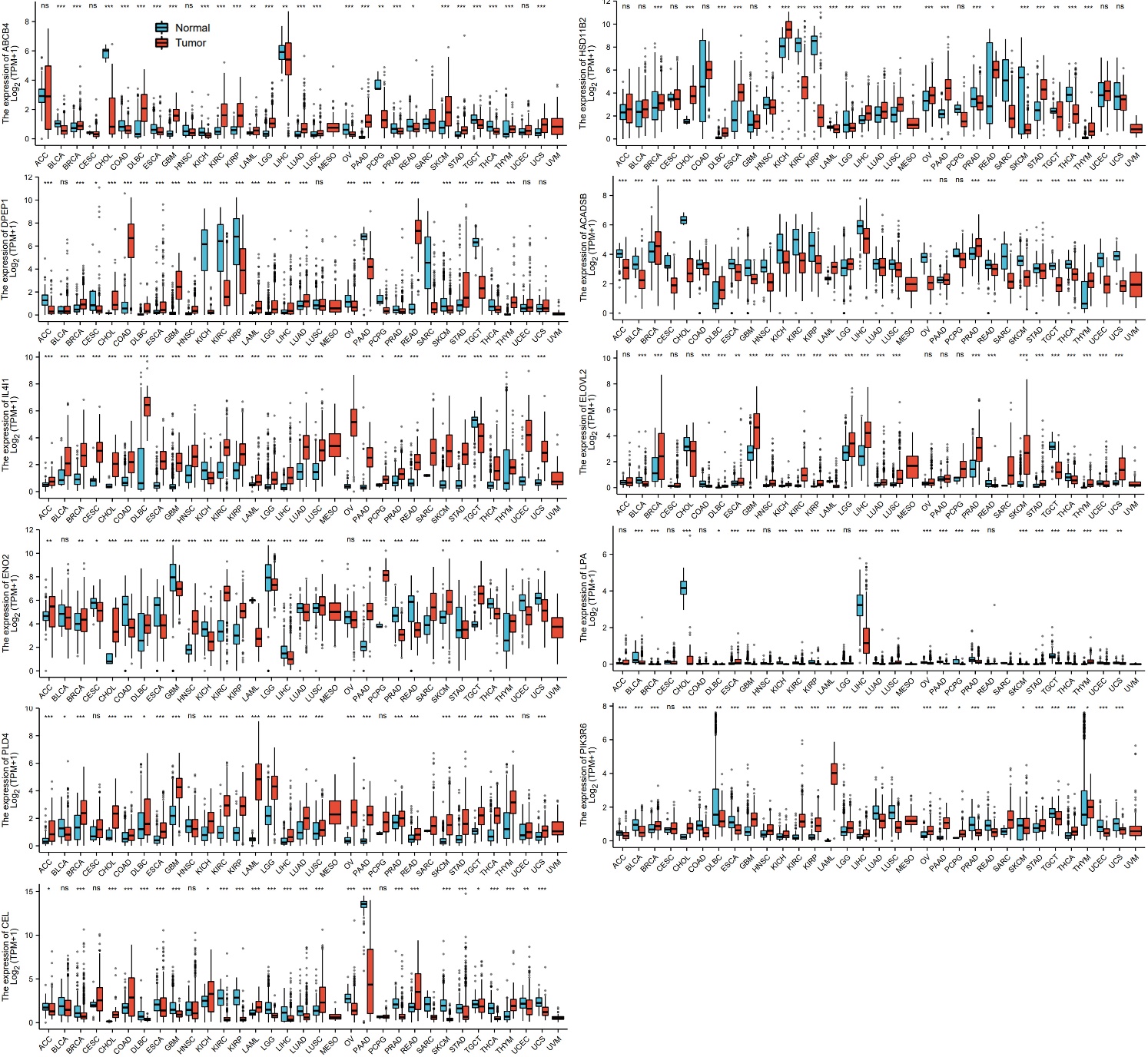


**Supplementary** **Figure 5.** Pan-cancer expression analysis of these eleven lipid metabolism genes (*ABCB4*, *DPEP1*, *IL4I1*, *ENO2*, *PLD4*, *CEL*, *HSD11B2*, *ACADSB*, *ELOVL2*, *LPA*, and *PIK3R6*). **p* < 0.05, ***p* < 0.01, ****p* < 0.001.

Supplementary Tables

**Supplementary Table 1. Lipid metabolism genes for filter**

| *AACS* | *AADAT* | *ABCA1* | *ABCB11* | *ABCB4* | *ABCC1* | *ABCC3* | *ABCD1* | *ABCG5* | *ABCG8* |
| --- | --- | --- | --- | --- | --- | --- | --- | --- | --- |
| *ABHD3* | *ABHD4* | *ABHD5* | *AC003665.1* | *AC092724.1* | *ACAA1* | *ACAA2* | *ACACA* | *ACACB* | *ACAD10* |
| *ACAD11* | *ACADL* | *ACADM* | *ACADS* | *ACADSB* | *ACADVL* | *ACAT1* | *ACAT2* | *ACBD4* | *ACBD5* |
| *ACBD6* | *ACBD7* | *ACER1* | *ACER2* | *ACER3* | *ACHE* | *ACLY* | *ACO2* | *ACOT1* | *ACOT11* |
| *ACOT12* | *ACOT13* | *ACOT2* | *ACOT4* | *ACOT6* | *ACOT7* | *ACOT8* | *ACOT9* | *ACOX1* | *ACOX2* |
| *ACOX3* | *ACOXL* | *ACP6* | *ACSBG1* | *ACSBG2* | *ACSF2* | *ACSF3* | *ACSL1* | *ACSL3* | *ACSL4* |
| *ACSL5* | *ACSL6* | *ACSM3* | *ACSM6* | *ACSS1* | *ACSS3* | *ADCY1* | *ADCY2* | *ADCY3* | *ADCY4* |
| *ADCY5* | *ADCY6* | *ADCY7* | *ADCY8* | *ADCY9* | *ADH1A* | *ADH1B* | *ADH1C* | *ADH4* | *ADH5* |
| *ADH6* | *ADH7* | *ADIPOQ* | *ADIPOR1* | *ADIPOR2* | *ADORA1* | *ADPRM* | *ADRB1* | *ADRB2* | *ADRB3* |
| *ADSL* | *AGK* | *AGMO* | *AGPAT1* | *AGPAT2* | *AGPAT3* | *AGPAT4* | *AGPAT5* | *AGPS* | *AGRP* |
| *AGT* | *AHR* | *AHRR* | *AKR1A1* | *AKR1B1* | *AKR1B10* | *AKR1B15* | *AKR1C1* | *AKR1C2* | *AKR1C3* |
| *AKR1C4* | *AKR1D1* | *AKT1* | *AKT2* | *AKT3* | *ALAD* | *ALAS1* | *ALB* | *ALDH1A1* | *ALDH1B1* |
| *ALDH2* | *ALDH3A1* | *ALDH3A2* | *ALDH3B1* | *ALDH3B2* | *ALDH7A1* | *ALDH9A1* | *ALDOA* | *ALOX12* | *ALOX12B* |
| *ALOX15* | *ALOX15B* | *ALOX5* | *ALOX5AP* | *ALOXE3* | *ALPI* | *AMACR* | *ANGPTL3* | *ANGPTL4* | *ANGPTL8* |
| *ANKRD1* | *AOC3* | *APEX1* | *APOA1* | *APOA2* | *APOA4* | *APOA5* | *APOB* | *APOC1* | *APOC2* |
| *APOC3* | *APOE* | *APOH* | *AQP7* | *ARF1* | *ARF3* | *ARNT* | *ARNT2* | *ARNTL* | *ARSA* |
| *ARSB* | *ARSD* | *ARSE* | *ARSF* | *ARSG* | *ARSH* | *ARSI* | *ARSJ* | *ARSK* | *ARV1* |
| *ASAH1* | *ASAH2* | *AUH* | *AWAT1* | *AWAT2* | *B3GALNT1* | *B4GALNT1* | *B4GALT6* | *BAAT* | *BCHE* |
| *BCKDHB* | *BDH1* | *BDH2* | *BLVRA* | *BMPR1B* | *BMX* | *BPHL* | *CA2* | *CA4* | *CA6* |
| *CAMKK2* | *CARM1* | *CAV1* | *CBR1* | *CBR3* | *CBR4* | *CCDC58* | *CCNC* | *CD1D* | *CD36* |
| *CDIPT* | *CDK19* | *CDK8* | *CDS1* | *CDS2* | *CEL* | *CEPT1* | *CERK* | *CERS1* | *CERS2* |
| *CERS3* | *CERS4* | *CERS5* | *CERS6* | *CETP* | *CGA* | *CH25H* | *CHAT* | *CHD9* | *CHKA* |
| *CHKB* | *CHPT1* | *CHUK* | *CIDEA* | *CIDEC* | *CLOCK* | *CLPS* | *COL4A3BP* | *COMT* | *CPNE1* |
| *CPNE3* | *CPNE6* | *CPNE7* | *CPOX* | *CPT1A* | *CPT1B* | *CPT1C* | *CPT2* | *CPTP* | *CRAT* |
| *CREBBP* | *CRLS1* | *CROT* | *CRYZ* | *CSNK1G2* | *CSNK2A1* | *CSNK2A2* | *CSNK2B* | *CTSA* | *CUBN* |
| *CYP11A1* | *CYP11B1* | *CYP11B2* | *CYP17A1* | *CYP19A1* | *CYP1A1* | *CYP1A2* | *CYP1B1* | *CYP21A2* | *CYP24A1* |
| *CYP27A1* | *CYP27B1* | *CYP2B6* | *CYP2C19* | *CYP2C8* | *CYP2C9* | *CYP2D6* | *CYP2E1* | *CYP2J2* | *CYP2R1* |
| *CYP2U1* | *CYP39A1* | *CYP3A4* | *CYP3A5* | *CYP3A7* | *CYP3A7-CYP3A51P* | *CYP46A1* | *CYP4A11* | *CYP4A22* | *CYP4B1* |
| *CYP4F11* | *CYP4F2* | *CYP4F22* | *CYP4F3* | *CYP4F8* | *CYP51A1* | *CYP7A1* | *CYP7B1* | *CYP8B1* | *D2HGDH* |
| *DBI* | *DDHD1* | *DDHD2* | *DECR1* | *DECR2* | *DEGS1* | *DEGS2* | *DGAT1* | *DGAT2* | *DGAT2L6* |
| *DGKA* | *DGKB* | *DGKD* | *DGKE* | *DGKG* | *DGKH* | *DGKI* | *DGKK* | *DGKQ* | *DGKZ* |
| *DHCR24* | *DHCR7* | *DHRS7B* | *DLD* | *DLST* | *DPEP1* | *DPEP2* | *DPEP3* | *EBP* | *ECH1* |
| *ECHS1* | *ECI1* | *ECI2* | *EHHADH* | *ELOVL1* | *ELOVL2* | *ELOVL3* | *ELOVL4* | *ELOVL5* | *ELOVL6* |
| *ELOVL7* | *ENO2* | *ENO3* | *ENPP2* | *ENPP6* | *ENPP7* | *EP300* | *EPHX1* | *EPHX2* | *ERP29* |
| *ESRRA* | *ESYT1* | *ESYT2* | *ESYT3* | *ETFDH* | *ETNK1* | *ETNK2* | *ETNPPL* | *FA2H* | *FAAH* |
| *FAAH2* | *FABP1* | *FABP12* | *FABP2* | *FABP3* | *FABP4* | *FABP5* | *FABP6* | *FABP7* | *FABP9* |
| *FADS1* | *FADS2* | *FAM120B* | *FAM213B* | *FAR1* | *FAR2* | *FASN* | *FDFT1* | *FDPS* | *FDX1* |
| *FDX2* | *FDXR* | *FH* | *FHL2* | *FIG4* | *FITM1* | *FITM2* | *FMO1* | *G0S2* | *G6PC* |
| *G6PC2* | *G6PC3* | *GABARAPL1* | *GAD2* | *GAL3ST1* | *GALC* | *GAPDHS* | *GBA* | *GBA2* | *GC* |
| *GCDH* | *GDE1* | *GDPD1* | *GDPD3* | *GDPD5* | *GGPS1* | *GGT1* | *GGT5* | *GK* | *GK2* |
| *GK3P* | *GLA* | *GLB1* | *GLB1L* | *GLIPR1* | *GLTP* | *GLUL* | *GLYCTK* | *GM2A* | *GNAI1* |
| *GNAI2* | *GNAI3* | *GNAS* | *GNPAT* | *GOT2* | *GPAM* | *GPAT2* | *GPAT3* | *GPAT4* | *GPCPD1* |
| *GPD1* | *GPD1L* | *GPD2* | *GPS2* | *GPX1* | *GPX2* | *GPX3* | *GPX4* | *GPX5* | *GPX6* |
| *GPX7* | *GPX8* | *GRHL1* | *GRHPR* | *GSTM4* | *GSTZ1* | *H2AFZ* | *HACD1* | *HACD2* | *HACD3* |
| *HACD4* | *HACL1* | *HADH* | *HADHA* | *HADHB* | *HAO2* | *HCCS* | *HDAC3* | *HELZ2* | *HEXA* |
| *HEXB* | *HIBCH* | *HILPDA* | *HMGCL* | *HMGCLL1* | *HMGCR* | *HMGCS1* | *HMGCS2* | *HPGD* | *HPGDS* |
| *HSD11B1* | *HSD11B2* | *HSD17B1* | *HSD17B10* | *HSD17B11* | *HSD17B12* | *HSD17B13* | *HSD17B14* | *HSD17B2* | *HSD17B3* |
| *HSD17B4* | *HSD17B6* | *HSD17B7* | *HSD17B8* | *HSD3B1* | *HSD3B2* | *HSD3B7* | *HSDL2* | *HSP90AA1* | *HSPH1* |
| *HTD2* | *IDH1* | *IDH3B* | *IDH3G* | *IDI1* | *IDI2* | *IKBKB* | *IKBKG* | *IL4I1* | *ILK* |
| *INMT* | *INPP4A* | *INPP4B* | *INPP5D* | *INPP5E* | *INPP5F* | *INPP5J* | *INPP5K* | *INPPL1* | *INS* |
| *INSIG1* | *INSIG2* | *INSR* | *IRS1* | *IRS2* | *IRS4* | *JAK2* | *JMJD7-PLA2G4B* | *KDSR* | *KMT5A* |
| *KPNB1* | *LBR* | *LCAT* | *LCLAT1* | *LDHA* | *LDLR* | *LDLRAP1* | *LEP* | *LEPR* | *LGALS1* |
| *LGMN* | *LHB* | *LIPA* | *LIPC* | *LIPE* | *LIPF* | *LIPG* | *LIPH* | *LIPI* | *LPA* |
| *LPCAT1* | *LPCAT2* | *LPCAT3* | *LPCAT4* | *LPGAT1* | *LPIN1* | *LPIN2* | *LPIN3* | *LPL* | *LRP1* |
| *LRP2* | *LRPAP1* | *LRTOMT* | *LSS* | *LTA4H* | *LTC4S* | *LYPLA1* | *LYPLA2* | *MAOA* | *MAPK10* |
| *MAPK8* | *MAPK9* | *MAPKAPK2* | *MBOAT1* | *MBOAT2* | *MBOAT7* | *MBTPS1* | *MBTPS2* | *MCAT* | *MCEE* |
| *MDH1* | *MDH2* | *ME1* | *MECR* | *MED1* | *MED10* | *MED11* | *MED12* | *MED13* | *MED13L* |
| *MED14* | *MED15* | *MED16* | *MED17* | *MED18* | *MED19* | *MED20* | *MED21* | *MED22* | *MED23* |
| *MED24* | *MED25* | *MED26* | *MED27* | *MED28* | *MED29* | *MED30* | *MED31* | *MED4* | *MED6* |
| *MED7* | *MED8* | *MED9* | *METAP1* | *MFSD2A* | *MGLL* | *MID1IP1* | *MIF* | *MIGA1* | *MIGA2* |
| *MLYCD* | *MMAA* | *MMP1* | *MMUT* | *MOGAT1* | *MOGAT2* | *MOGAT3* | *MORC2* | *MSMO1* | *MTF1* |
| *MTM1* | *MTMR1* | *MTMR10* | *MTMR12* | *MTMR14* | *MTMR2* | *MTMR3* | *MTMR4* | *MTMR6* | *MTMR7* |
| *MTMR8* | *MTMR9* | *MTOR* | *MTTP* | *MVD* | *MVK* | *MYLIP* | *NBN* | *NCAPH2* | *NCEH1* |
| *NCOA1* | *NCOA2* | *NCOA3* | *NCOA6* | *NCOR1* | *NCOR2* | *NDUFAB1* | *NEU1* | *NEU2* | *NEU3* |
| *NEU4* | *NFKB1* | *NFKBIA* | *NFKBIB* | *NFKBIE* | *NFYA* | *NFYB* | *NFYC* | *NPAS2* | *NPC1* |
| *NPC1L1* | *NPC2* | *NPR1* | *NPY* | *NPY1R* | *NR1D1* | *NR1H2* | *NR1H3* | *NR1H4* | *NRF1* |
| *NSDHL* | *NTHL1* | *NUDT19* | *OCRL* | *ODC1* | *OLAH* | *OLR1* | *ORMDL1* | *ORMDL2* | *ORMDL3* |
| *OSBP* | *OSBPL10* | *OSBPL1A* | *OSBPL2* | *OSBPL3* | *OSBPL5* | *OSBPL6* | *OSBPL8* | *OSBPL9* | *OSTC* |
| *OXCT1* | *OXCT2* | *OXSM* | *PAFAH1B1* | *PAFAH1B2* | *PAFAH1B3* | *PAFAH2* | *PCBD1* | *PCCA* | *PCCB* |
| *PCK1* | *PCK2* | *PCSK9* | *PCTP* | *PCYT1A* | *PCYT1B* | *PCYT2* | *PDE3B* | *PDHA1* | *PDHB* |
| *PDPK1* | *PECR* | *PEMT* | *PEX11A* | *PGS1* | *PHOSPHO1* | *PHYH* | *PI4K2A* | *PI4K2B* | *PI4KA* |
| *PI4KB* | *PIAS4* | *PIK3C2A* | *PIK3C2B* | *PIK3C2G* | *PIK3C3* | *PIK3CA* | *PIK3CB* | *PIK3CD* | *PIK3CG* |
| *PIK3R1* | *PIK3R2* | *PIK3R3* | *PIK3R4* | *PIK3R5* | *PIK3R6* | *PIKFYVE* | *PIP4K2A* | *PIP4K2B* | *PIP4K2C* |
| *PIP4P1* | *PIP5K1A* | *PIP5K1B* | *PIP5K1C* | *PISD* | *PITPNB* | *PITPNM1* | *PITPNM2* | *PITPNM3* | *PLA1A* |
| *PLA2G10* | *PLA2G12A* | *PLA2G12B* | *PLA2G15* | *PLA2G16* | *PLA2G1B* | *PLA2G2A* | *PLA2G2C* | *PLA2G2D* | *PLA2G2E* |
| *PLA2G2F* | *PLA2G3* | *PLA2G4A* | *PLA2G4B* | *PLA2G4C* | *PLA2G4D* | *PLA2G4E* | *PLA2G4F* | *PLA2G5* | *PLA2G6* |
| *PLA2G7* | *PLA2R1* | *PLAAT1* | *PLAAT2* | *PLAAT3* | *PLAAT4* | *PLAAT5* | *PLB1* | *PLBD1* | *PLD1* |
| *PLD2* | *PLD3* | *PLD4* | *PLD6* | *PLEKHA1* | *PLEKHA2* | *PLEKHA3* | *PLEKHA4* | *PLEKHA5* | *PLEKHA6* |
| *PLEKHA8* | *PLIN1* | *PLIN2* | *PLIN3* | *PLIN4* | *PLIN5* | *PLPP1* | *PLPP2* | *PLPP3* | *PLPP4* |
| *PLPP5* | *PLPP6* | *PLTP* | *PMVK* | *PNLIP* | *PNLIPRP1* | *PNLIPRP2* | *PNLIPRP3* | *PNPLA2* | *PNPLA3* |
| *PNPLA4* | *PNPLA5* | *PNPLA6* | *PNPLA7* | *PNPLA8* | *POMC* | *PON1* | *PON2* | *PON3* | *PPARA* |
| *PPARD* | *PPARG* | *PPARGC1A* | *PPARGC1B* | *PPM1L* | *PPP1CA* | *PPP1CB* | *PPP1CC* | *PPT1* | *PPT2* |
| *PRDX6* | *PRKAA1* | *PRKAA2* | *PRKAB1* | *PRKAB2* | *PRKACA* | *PRKACB* | *PRKACG* | *PRKAG1* | *PRKAG2* |
| *PRKAG3* | *PRKCQ* | *PRKD1* | *PRKD2* | *PRKD3* | *PRKG1* | *PRKG2* | *PRXL2B* | *PSAP* | *PSME1* |
| *PTDSS1* | *PTDSS2* | *PTEN* | *PTGDS* | *PTGER3* | *PTGES* | *PTGES2* | *PTGES3* | *PTGIS* | *PTGR1* |
| *PTGR2* | *PTGS1* | *PTGS2* | *PTPMT1* | *PTPN11* | *PTPN13* | *PTPRG* | *PTS* | *RAB14* | *RAB4A* |
| *RAB5A* | *RAN* | *RAP1GDS1* | *RDH11* | *RDH16* | *REEP6* | *RELA* | *RETSAT* | *RGL1* | *RORA* |
| *RUFY1* | *RXRA* | *RXRB* | *RXRG* | *S100A10* | *SACM1L* | *SAMD8* | *SAR1B* | *SBF1* | *SBF2* |
| *SC5D* | *SCAP* | *SCARB1* | *SCD* | *SCD5* | *SCP2* | *SDHA* | *SDHC* | *SDHD* | *SEC23A* |
| *SEC24A* | *SEC24B* | *SEC24C* | *SEC24D* | *SELENOI* | *SERINC1* | *SGMS1* | *SGMS2* | *SGPL1* | *SGPP1* |
| *SGPP2* | *SIN3A* | *SIN3B* | *SLC10A1* | *SLC10A2* | *SLC22A5* | *SLC25A1* | *SLC25A17* | *SLC25A20* | *SLC27A1* |
| *SLC27A2* | *SLC27A3* | *SLC27A4* | *SLC27A5* | *SLC27A6* | *SLC2A1* | *SLC2A4* | *SLC44A1* | *SLC44A2* | *SLC44A3* |
| *SLC44A4* | *SLC44A5* | *SLCO1A2* | *SLCO1B1* | *SLCO1B3* | *SMARCD3* | *SMPD1* | *SMPD2* | *SMPD3* | *SMPD4* |
| *SMS* | *SOAT1* | *SOAT2* | *SOCS3* | *SORBS1* | *SORT1* | *SP1* | *SPHK1* | *SPHK2* | *SPTLC1* |
| *SPTLC2* | *SPTLC3* | *SPTSSA* | *SPTSSB* | *SQLE* | *SRD5A1* | *SRD5A2* | *SRD5A3* | *SREBF1* | *SREBF2* |
| *STAR* | *STARD10* | *STARD3* | *STARD3NL* | *STARD4* | *STARD5* | *STARD6* | *STARD7* | *STAT3* | *STK11* |
| *STS* | *SUCLA2* | *SUCLG1* | *SUCLG2* | *SULT1E1* | *SULT2A1* | *SULT2B1* | *SUMF1* | *SUMF2* | *SUMO2* |
| *SYNJ1* | *SYNJ2* | *TAZ* | *TBL1X* | *TBL1XR1* | *TBXAS1* | *TDO2* | *TECR* | *TECRL* | *TGS1* |
| *THEM4* | *THEM5* | *THRAP3* | *THRSP* | *TIAM2* | *TKFC* | *TM7SF2* | *TMEM86B* | *TNF* | *TNFAIP8* |
| *TNFAIP8L1* | *TNFAIP8L2* | *TNFAIP8L3* | *TNFRSF1A* | *TNFRSF1B* | *TNFRSF21* | *TP53INP2* | *TPTE* | *TPTE2* | *TRADD* |
| *TRAF2* | *TRIB3* | *TSHB* | *TSHR* | *TSPO* | *TSPOAP1* | *TXNRD1* | *UBC* | *UBE2I* | *UBE2L6* |
| *UCP1* | *UGCG* | *UGDH* | *UGT1A1* | *UGT1A10* | *UGT1A3* | *UGT1A4* | *UGT1A5* | *UGT1A6* | *UGT1A7* |
| *UGT1A8* | *UGT1A9* | *UGT2A1* | *UGT2A2* | *UGT2A3* | *UGT2B10* | *UGT2B11* | *UGT2B15* | *UGT2B17* | *UGT2B28* |
| *UGT2B4* | *UGT2B7* | *UGT8* | *UROD* | *UROS* | *VAC14* | *VAPA* | *VAPB* | *VDAC1* | *VDAC2* |
| *VDAC3* | *VDR* | *VNN1* | *XIST* | *YWHAH* |  |  |  |  |  |

**Supplementary Table 2. Univariate Cox regression analysis in the TCGA-KIRC**

| **Characteristics** | **HR** | **95% CI** | ***p*-value** |
| --- | --- | --- | --- |
| *ABCB4* | 0.708 | 0.596-0.842 | 0.000† |
| *ALOX5* | 1.122 | 0.999-1.260 | 0.051† |
| *DPEP1* | 0.848 | 0.770-0.935 | 0.001† |
| *PTGER3* | 0.853 | 0.784-0.927 | 0.000† |
| *SCARB1* | 0.930 | 0.832-1.039 | 0.199 |
| *APOB* | 1.051 | 0.957-1.154 | 0.294 |
| *APOH* | 1.089 | 0.997-1.190 | 0.057† |
| *SCD* | 1.020 | 0.903-1.153 | 0.747 |
| *LGALS1* | 1.484 | 1.251-1.761 | 0.000† |
| *CPNE6* | 0.427 | 0.128-1.431 | 0.168 |
| *PCK2* | 0.716 | 0.599-0.856 | 0.000† |
| *TRIB3* | 1.344 | 1.195-1.510 | 0.000† |
| *IL4I1* | 1.230 | 1.061-1.426 | 0.006† |
| *CAV1* | 1.010 | 0.863-1.182 | 0.905 |
| *ABCC3* | 1.053 | 0.923-1.201 | 0.442 |
| *CYP27B1* | 1.181 | 0.979-1.423 | 0.081† |
| *ENO2* | 1.192 | 1.037-1.371 | 0.014† |
| *REEP6* | 1.169 | 1.001-1.365 | 0.048† |
| *HAO2* | 0.827 | 0.772-0.885 | 0.000† |
| *PLA2G2D* | 1.131 | 1.014-1.262 | 0.027† |
| *PCK1* | 0.837 | 0.784-0.894 | 0.000† |
| *GAL3ST1* | 0.959 | 0.873-1.054 | 0.385 |
| *APOC1* | 1.118 | 1.011-1.236 | 0.029† |
| *G6PC* | 0.755 | 0.681-0.837 | 0.000† |
| *ALDH3B2* | 1.120 | 0.763-1.645 | 0.562 |
| *HMGCS2* | 0.844 | 0.791-0.900 | 0.000† |
| *CYP2J2* | 0.885 | 0.834-0.938 | 0.000† |
| *CD36* | 0.834 | 0.754-0.922 | 0.000† |
| *B4GALNT1* | 1.073 | 0.982-1.173 | 0.118 |
| *BAAT* | 0.996 | 0.879-1.128 | 0.949 |
| *BMPR1B* | 1.063 | 0.953-1.186 | 0.271 |
| *PIK3C2G* | 0.874 | 0.607-1.259 | 0.471 |
| *PIK3R5* | 0.999 | 0.837-1.193 | 0.993 |
| *PLA2G7* | 0.920 | 0.817-1.035 | 0.165 |
| *PLIN2* | 0.870 | 0.802-0.943 | 0.001† |
| *PLA2R1* | 0.917 | 0.767-1.096 | 0.341 |
| *LPCAT1* | 1.056 | 0.918-1.216 | 0.444 |
| *CD1D* | 0.882 | 0.735-1.058 | 0.177 |
| *CYP3A4* | 0.490 | 0.296-0.812 | 0.006† |
| *TNFAIP8L2* | 1.194 | 0.999-1.427 | 0.051† |
| *FABP1* | 0.879 | 0.770-1.004 | 0.056† |
| *ALB* | 0.946 | 0.832-1.077 | 0.403 |
| *LIPH* | 1.070 | 0.949-1.205 | 0.269 |
| *ENPP6* | 0.824 | 0.614-1.105 | 0.195 |
| *ACSL6* | 0.814 | 0.566-1.169 | 0.264 |
| *FABP7* | 1.019 | 0.973-1.068 | 0.424 |
| *TSHR* | 0.912 | 0.676-1.230 | 0.546 |
| *MOGAT2* | 1.260 | 0.689-2.303 | 0.454 |
| *PLD4* | 0.823 | 0.704-0.962 | 0.014† |
| *ACSF2* | 1.077 | 0.911-1.272 | 0.387 |
| *DPEP2* | 0.913 | 0.736-1.134 | 0.411 |
| *ANGPTL4* | 0.966 | 0.896-1.043 | 0.378 |
| *PLA2G4F* | 0.866 | 0.721-1.041 | 0.126 |
| *FABP6* | 1.058 | 0.968-1.157 | 0.217 |
| *CEL* | 1.229 | 1.048-1.440 | 0.011† |
| *ACOT12* | 0.657 | 0.299-1.443 | 0.295 |
| *ADH6* | 0.755 | 0.659-0.864 | 0.000† |
| *UGT2A1* | 0.907 | 0.555-1.481 | 0.696 |
| *HSD11B2* | 0.787 | 0.713-0.868 | 0.000† |
| *ALOX15B* | 0.966 | 0.851-1.096 | 0.590 |
| *CYP4F2* | 1.007 | 0.860-1.178 | 0.934 |
| *CYP4F3* | 1.138 | 1.020-1.268 | 0.020† |
| *ACADSB* | 0.582 | 0.501-0.676 | 0.000† |
| *MMP1* | 1.117 | 1.027-1.215 | 0.010† |
| *ADH1B* | 0.976 | 0.892-1.067 | 0.593 |
| *CYP2B6* | 0.902 | 0.690-1.179 | 0.450 |
| *ELOVL2* | 1.120 | 0.983-1.276 | 0.088† |
| *LPA* | 0.162 | 0.052-0.500 | 0.002† |
| *ACOT6* | 0.946 | 0.599-1.493 | 0.810 |
| *ADH1C* | 0.975 | 0.824-1.153 | 0.765 |
| *PIK3R6* | 1.341 | 1.145-1.571 | 0.000† |

HR, hazard ratio; CI, confidence interval; †*p* < 0.10.
